# Tetrodotoxin: Chemistry, Toxicity, Source, Distribution and Detection

**DOI:** 10.3390/toxins6020693

**Published:** 2014-02-21

**Authors:** Vaishali Bane, Mary Lehane, Madhurima Dikshit, Alan O’Riordan, Ambrose Furey

**Affiliations:** 1 Mass Spectrometry Research Centre (MSRC) and PROTEOBIO Research Groups, Department of Chemistry, Cork Institute of Technology, Rossa Avenue, Bishopstown, Cork, Ireland; E-Mails: vaishali.p.bane@cit.ie (V.B.); mary.lehane@cit.ie (M.L.); 2 Department of Chemistry, University of Pune, Pune 411007, India; E-Mail: mdikshit@chem.unipune.ernet.in; 3 Nanotechnology Group, Tyndall National Institute, University College Cork, Lee Maltings, Cork, Ireland; E-Mail: alan.oriordan@tyndall.ie

**Keywords:** tetrodotoxin, TTX, TTX analogues, chemistry, toxicity, source, distribution, detection

## Abstract

Tetrodotoxin (TTX) is a naturally occurring toxin that has been responsible for human intoxications and fatalities. Its usual route of toxicity is via the ingestion of contaminated puffer fish which are a culinary delicacy, especially in Japan. TTX was believed to be confined to regions of South East Asia, but recent studies have demonstrated that the toxin has spread to regions in the Pacific and the Mediterranean. There is no known antidote to TTX which is a powerful sodium channel inhibitor. This review aims to collect pertinent information available to date on TTX and its analogues with a special emphasis on the structure, aetiology, distribution, effects and the analytical methods employed for its detection.

## 1. Introduction

In Japan *fugu* or puffer fish, is a long established delicacy, in spite of its known potential for toxicity. Tetrodotoxin (TTX) is the naturally occurring toxin that is mainly responsible for the risk to consumers [1,2,3,4,5,6,7,8,9,10,11,12,13]. In fact, over 20 species of puffer fish have been found to harbour the toxin [14]. TTX is both water soluble and heat stable so cooking does not negate its toxicity; rather it increases toxic effect [15]. Consequently, great care must be taken by specially trained chefs to ensure that the hazardous parts of the fish (specifically the liver, ovaries and skin) are excised before serving; nevertheless human intoxications and some fatalities have been linked to the consumption of puffer fish [1,2,3,6,9,11,16,17,18,19].

The toxin was first discovered in 1909 by Dr. Yoshizumi Tahara from the ovaries of globefish [20], but puffer fish have been known to be toxic to humans for a long time. TTX is a very potent neurotoxin that is found in a variety of marine [3,5,10,21,22,23,24,25,26,27,28,29] and also in some terrestrial [30,31,32,33,34,35,36,37] species. Its toxicity is often emphasised by referring to the fact that it is over a thousand times more toxic to humans than cyanide; TTX has no known antidote [38,39]. 

Besides puffer fish, other species known to harbour TTX include: gastropods [4,5,7,8,10,12,13,25,28,40,41,42,43,44,45,46,47,48], newts [31,32,35,36,37,49,50,51,52,53,54,55,56,57,58,59,60], crabs [61,62,63,64,65,66,67,68,69], frogs [30,33,34,70,71,72], sea slugs [73,74], star fishes [75], blue-ringed octopuses [76,77,78], ribbon worms [22,79] and bacteria [74,80,81,82,83,84,85,86,87,88]. The distribution of TTX and its analogues are known to be organism and/or tissue specific [46].

There is some conjecture as to the origin of TTX in puffer fish. It has been shown that cultured non-toxic puffer fish when fed on a diet containing TTX become toxic [89] and toxic puffer fish when fed on a TTX-free diet become non-toxic [90]. It has also been demonstrated that the source of TTX in puffer fish is an endo-symbiotic bacteria that naturally inhabits the gut of the animal [81,82,87,88,91,92]. This may be explained by the hypothesis that puffer fish could initially acquire the TTX producing bacteria via the food web and that these bacteria then persist in the fish [93]. Indeed several ubiquitous varieties of bacteria produce TTX including some in the *Pseudomonas* and *Vibrio* genera [21,80,81,82,83,84,85,86,87,88,91,92,94,95]. Origin of TTX in terrestrial animal’s, for example newt and frog is endogenous because it has role in defense from predators in these animals (Refer to paragraph 4.3). 

The mechanism of TTX toxicity has been investigated in animal models [15,96,97]: It is a sodium channel blocker. The toxin binds to the sodium channels of the excitable tissues of the victim (muscles and nerves); the inhibition of sodium ions through the channels effectively immobilises these tissues [98]. In humans the onset and severity of the symptoms of TTX poisoning after ingestion is dose dependent [19]. Initial symptoms include tingling (paresthesias) of the tongue and lips, followed by or concurrent with headache and vomiting, which may progress to muscle weakness and ataxia. In severe cases death may occur due to respiratory and/or heart failure [6]. The only treatment for TTX intoxication is observation and appropriate supportive care [38].

TTX was regarded until the recent past as a problem confined to Japan and Asian countries; now the problem is emerging as a threat to regions that were here to fore considered safe, refer to Table 1. It is thought that the “spread” of the toxin is due to increasing water temperatures world-wide [99]. In this review we will examine reports regarding the geographic distribution of TTX (Table 1).

**Table 1:** Worldwide occurrence of tetrodotoxin (TTX) poisoning.

Country	Causative Organism	Analogues of TTX found	No. of cases	Year of poisoning incident	Reference
Australia	Toadfish	TTX	7	2004	[100]
Australia	Puffer fish	TTX	11	1 January 2001 to 13 April 2002	[101]
Bangladesh	Puffer fish, Dora potka *i.e.*, *Takifugu oblongus* in Natore District and Badami potka *i.e.*, *Arothron stellatus* in Narsingdi District	TTX	141 (Three outbreaks) 48 (Narsingdi District) + 10 (Dhaka) + 83 (Natore District)	9 April 2008 (Narsingdi District) 3 June 2008 (Dhaka) 8 June 2008 (Natore District)	[19]
Bangladesh	Puffer fish	TTX	53	May 2001–May 2006	[102]
Bangladesh (Khulna district)	Puffer fish	TTX	37 (8 died)	April 2002	[9]
Bangladesh (Degholia in the Khulna district)	Puffer fish, *Takifugu oblongus*	TTX	36 (7 died)	May 2002	[11]
Bangladesh	Puffer fish, *Takifugu oblongus*	TTX	8 (5 died)	1998	[3]
China (Lianyungang)	Gastropod, *Nassarius* spp.	TTX, trideoxyTTX, 4-epiTTX, anhydroTTX and oxoTTX	-	May–August 2007	[28]
China (South Zheijiang, Mainland)	Gastropod, *Zeuxis samiplicutus*	TTX	30	June 2001	[5]
India (Orissa state, Burla)	Puffer fish	TTX	8	October 2007	[17]
Japan	–	TTX	Numerous	1965–2010	[46]
Japan	Puffer fish “kinfugu”, *T. poecilonotus*	TTX	1	October 2008	[103]
Japan	Thread-sail filefish “Kawahagi”	TTX	1	October 2008	[103]
Japan	Marine snail, *Nassarius glans*	TTX	1	July 2007	[103]
Japan	Marine snail, *C. saulie*	TTX	2	December 1987	[46]
Japan	Marine snail, *C. saulie*	TTX	1	December 1979	[40]
Japan	Marine snail (Ivory shell), *Babylonia japonica*	TTX	5	June 1957	[16]
Korea	Unknown fish	TTX	3	October 2010	[104]
Mediterranean region (Egypt and Israel)	Puffer fish, *L. sceleratus*	TTX	13 (9) Hafia bay, (2) Caesarea coast, (2) Ashkelon coast	November 2005, February 2007, March 2007, November 2007, March 2008 and May 2008	[105]
New Zealand	Grey side-gilled sea slug, *Pleurobranchaea maculata*	TTX	15 dogs	July to November 2009	[46]
Spain (Malaga; caught from Portuguese waters)	Trumpet shell, *Choronia lampus*	TTX	–	October 2007	[45]
Taiwan (Kaohsiung)	Gastropod, *Niotha clathrata*	TTX	3	November 2006	[13]
Taiwan	Gastropod	TTX and PSP	1	October 2005	[12]
Taiwan (Southern China Sea)	Marine snail, *Nassarius* (*Alectricon*) *glans*	TTX	5	April 2004	[46]
Taiwan (Tungsa Island)	Gastropod, *Nassarius glans*	TTX	6	April 2004	[10]
Taiwan (Western)	Gastropod, *Polinices didyma* and *Natica lineata*	TTX	–	2003	[8]
Taiwan (Tungkang, Southern Taiwan)	Gastropods, *Oliva miniacea*, *Oliva mustelina* and *Oliva nirasei*	TTX	1	February 2002	[7]
Taiwan	Unknown fish	TTX	6 (1 died)	April 2001	[106]
Taiwan	Puffer fish, *Lagocephalus lunaris*	TTX	6 (1 died)	April 2001	[6]
Taiwan (Northern)	Gastropods (snails), *Zeuxis sufflatus* and *Niotha clathrata*	TTX	4	April 2001	[4]
Taiwan (Chunghua Prefecture, Western Taiwan)	Puffer fish, *Takifugu niphobles*	TTX	5	Jan 2000	[107]
Thailand (Chon Buri, Eastern Thailand)	Eggs of horseshoe crab, *Carcinoscorpius rotundicauda*	TTX	71	1995	[63]
US (New Hampshire, New York, Pennsylvania and Virginia)	Newt, *N. viridescens*	TTX, 6-epiTTX and 11-oxoTTX	Collected samples for analysis	2001–2009	[37]
US (Chicago)	Puffer fish	TTX	2	May 2007	[108]
US (California)	Puffer fish transported from Japan	TTX	3	April 1996	[109]
US (Hawaii)	Puffer fish, *Diodon hystrix*	TTX	1	1986	[110]

**Figure 1:**
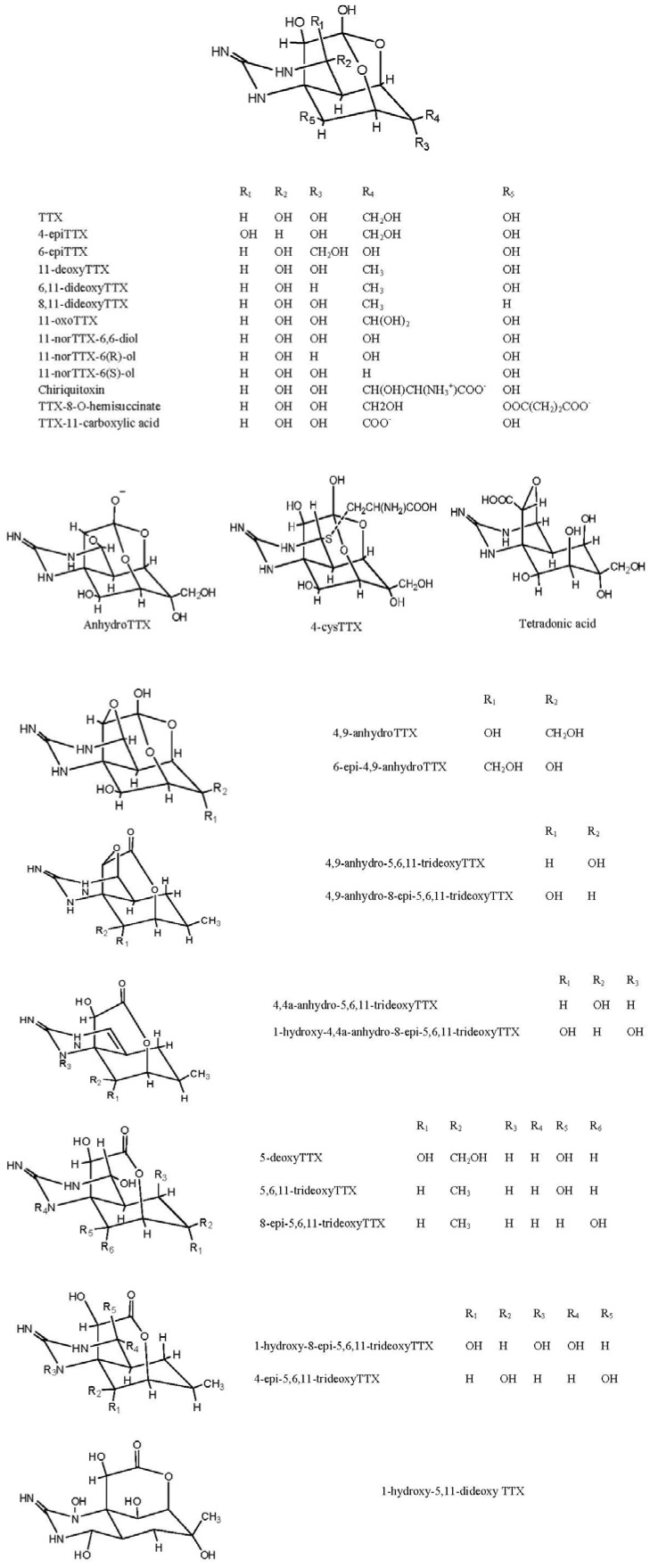
Structures of TTX analogues.

Structurally TTX consists of a guanidinium moiety connected to a highly oxygenated carbon skeleton that possesses a 2,4-dioxaadamantane portion containing five hydroxyl groups [111]. TTX co-exists with its naturally occurring analogues. There have been 30 structural analogues of TTX reported to date (Figure 1) and the degree of toxicity varies with structure [112]. One of the major problems for studying these analogues is the lack of commercially available standards. A number of researcher groups have synthesized some of the analogues of TTX on a laboratory scale [113,114,115,116,117,118,119,120] but availability is severely limited. 

Several analytical methods have been used for the detection and quantitation of TTX and its analogues. Mouse bioassay is commonly used for TTX analysis detecting TTX [27,121,122,123] and some of its toxic analogues (5,6,11-trideoxyTTX [124], 11-deoxyTTX [31] and 6,11-dideoxyTTX [125]). Liquid chromatography hyphenated with mass spectrometry (LC-MS/MS) detection is the preferred method for analysis of TTX, due to its unrivalled selectivity [13,25,106,126,127,128]. Toxicity limits for TTX in mice had been established [31,125,129,130], but there is still a need to establish accurate regulatory limits for TTX in humans [46,131]. However, the shortage of standards for the TTX analogues is the main hindrance for such studies to be undertaken.

It is important to analyse TTX analogues for toxicity because extensive toxicity studies have not been conducted on all the analogues. It is known that the deoxy analogues of TTX are less toxic than TTX, but the hydroxyl analogues are more toxic than TTX, this is likely to have implications in risk analysis studies concerning the effect of the toxin family on human health, please refer to Section 9 for a more in-depth discussion.

## 2. Structures and Standards for TTX and Its Analogues

The name TTX was coined by Dr. Yoshizumi Tahara in 1909. He isolated TTX from the ovaries of globefish [132]. TTX is a heterocyclic, organic perhydroquinozolineamine molecule (aminoperhydroquinazolone). Its structure was elucidated by R. B. Woodward in 1964 [133]. Authentic standards for TTX are available from various commercial sources (Table 2); Wako Chemicals (Purity >90%) [134], Latoxan (Valence, France) [43], Sigma Aldrich (St. Louis, MO, USA), 99% HPLC [104,135], Sankyo Co., Ltd, Tokyo, Japan [136], Calbiochem, EMD Millipore, USA, [51], Ascent Scientific [137] and Axxora, Grunberg, Germany [138]. 

All of these suppliers obtain TTX from TTX containing organisms (most commonly puffer fish ovary). These standards sometimes contain other analogues of TTX depending upon the source organism. O’Leary *et al*., 2004 stated that they had found two peaks in LC-MS analysis of standards for TTX supplied by Sigma-Aldrich, Australia as well as with Latoxan standards [120]. They assumed that the minor peak is anhydro-TTX. Therefore most standards are certified only for TTX (except for Wako Chemicals who supply standards for TTX, 4-epiTTX and anhydroTTX [139]). Though other analogues are present in the vast majority of standards they cannot be accurately quantified against a certified material standard, as there is a lack of analogue standards. 

There are 26 naturally occurring analogues of TTX (Table 3). As the authentic standards for these analogues are not commercially available, they have to be extracted and isolated from available sources (with further chemical modification in some cases); TTX was isolated from the ribbon worm, *Cephalothrix simula* (formerly *Cephalothrix* sp.) along with 4-epiTTX and 4,9-anhydroTTX [22,40,141]; TTX from the ovaries and the livers of puffer fish, *Fugu Pardalis* [24]; 11-deoxy TTX from the newt, *Cynops ensicauda* [31]; TTX, 4-epiTTX and 4,9-anhydroTTX from the eggs of the puffer fishes, *Fugu poecilonotus* and *Fugu pardalis* [142]; 5,6,11-TrideoxyTTX from the eggs of *F. poecilonotus* [124]; 6-epiTTX and 11-deoxyTTX from the newt, *Cynops ensicauda* [31]; Chiriquitoxin from the frog, *Atelopus chiriquiensis* [143]; TTX, 4-epi TTX and anhydro TTX from the liver of puffer fish, *Takifugu oblongus* [8]; TTX, 4-epi TTX, 4,9-anhydro TTX and 5,6,11-trideoxy TTX from the puffer fishes, *Fugu poecilonotus* [124] and *Fugu Pardalis* [124]; TTX (94.3% purity) from the liver of puffer, *Takifugu oblongus* by the method of Hwang *et al*., 1988 [106,144]; 6-epi TTX and 11-deoxy TTX from the newt, *Cynops ensicauda* [31] and TTX, 6-epi TTX, 4-epi TTX and 4,9-anhydro TTX from the puffer fish, *Spheroids Spengler* by the method of Goto *et al*., 1965 [145]. 11-norTTX-6(S)-ol, 11-norTTX-6(R)-ol and 11-norTTX-6,6-diol have been synthesized [146,147,148,149]. 

Yotsu-Yamashita *et al*., 1999 synthesized eight derivatives of TTX by chemical modifications of TTX extracted from the puffer fishes, *Fugu poecilonotus* and *Fugu pardalis*: 11-oxoTTX, TTX-11-carboxylic acid, 11-norTTX-6,6-diol, 11-norTTX-6(S)-ol, 11-norTTX-6(R)-ol, tetrodonic acid, 4,9-anhydro-8-O-hemisaccinate and TTX-8-O-hemisaccinate. Though these were just one or two step chemical reactions, the percentage yields of each derivative were 5%, 7%, 73%, 14%, 4%, 75%, 40% and 34% respectively [112].

Four new analogues, 8-epi-5,6,11-trideoxyTTX, 4,9-anhydro-8-epi-5,6,11-trideoxyTTX, 1-hydroxy-8-epi-5,6,11-trideoxyTTX and 1-hydroxy-4,4a-anhydro-8-epi-5,6,11-trideoxyTTX were recently isolated from newt, *Cynops ensicauda popei by* Kudo *et al*., 2012 [59]. 

Apart from the commercially available standards given, very few of the isolated TTX analogues or derivatives listed, are available in sufficient quantities to allow for the development and implementation of widespread screening by quantitative analytical methods.

**Table 2:** Commercial sources for TTX.

No.	Name of TTX analogue	Source for extraction	Purity (%)	CAS No.	Contact details
1	Tetrodotoxin (citrate free)	*Fugu* fish organs	96 (HPLC‚ IR‚ NMR)	4368-28-9	[150]
2	Tetrodotoxin (citrate free)	*Fugu* spp.	100	4368-28-9	[151]
3	Tetrodotoxin (citrate free)	NM	NM	4368-28-9	[152]
4	Tetrodotoxin (citrate free)	NM	NM	4368-28-9	[153]
5	Tetrodotoxin (MW 328.28)	*Fugu* spp.	NM	4368-28-9	[154]
6	Tetrodotoxin (MW 319.27) C_11_H_17_N_3_O_8_	NM	≥98 (HPLC)	4368-28-9	[155]
7	Tetrodotoxin (citrate free), C_11_H_17_N_3_O_8_, MW 319.3	NM	NM	4368-28-9	[156]
8	Tetrodotoxin (citrate free), C_11_H_17_N_3_O_8_, MW 319.28	*Tetraodon pardalis*	>98	4368-28-9	[157]
9	Tetrodotoxin (citrate free), C_11_H_17_N_3_O_8, _MW 319.3	*Fugu* spp.	≥95 by TLC	4368-28-9	[158]
10	Tetrodotoxin (citrate free), C_11_H_17_N_3_O_8, _MW 319.2	NM	>98	4368-28-9	[159]
11	Tetrodotoxin citrate, C_11_H_17_N_3_O_8, _MW 319.27	*Fugu*	>98	18660-81-6	[160]
12	Tetrodotoxin citrate, C_11_H_17_N_3_O_8, _MW 319.27	NM	>98	4368-28-9	[161]
13	Tetrodotoxin (citrate free), C_11_H_17_N_3_O_8_, MW 319.27	NM	NM	4368-28-9	[162]
14	Tetrodotoxin (citrate free), C_11_H_17_N_3_O_8_, MW 319.3	NM	NM	4368-28-9	[163]

Note: NM, Not mentioned.

**Table 3:** Sources of TTX analogues.

No.	Analogue	Exact Molar Mass	Molecular Formula	MRM	Source Organism
1	4-S-Cys TTX	422.110753	C_14_H_22_N_4_O_9_S	NR	Puffer fish: *Fugu Pardalis* [24,164]
2	TTX-8-O-hemisuccinate	418.109787	C_15_H_20_N_3_O_11_**^−^**	NR	Synthetic analogue [112]
3	Chiriquitoxin	392.117945	C_13_H_20_N_4_O_10_	NR	Toad*: Atelopus chiriquiensis* [30,70,143]
4	11-oxoTTX	335.096482	C_11_H_17_N_3_O_9_	336/318 336/300 336/282 336/178 336/162	(i) Puffer fish: *Arothron nigropunctatus* [165] (ii) Gastropod: *Nassarius* spp. [28] (iii) Xanthid crab: *Atergatis floridus* [62] (iv) Newt: *Taricha* spp. [36] and *Notophthalmus viridescens* [36,37,53] (v) Frog: *Brachycephalus ephippium* [34] and *B. pernix* [72]
5	TTX-11-carboxylic acid	332.073007	C_11_H_14_N_3_O_9_^−^	NR	Synthetic analogue [112]
6	TTX	319.101567	C_11_H_17_N_3_O_8_	320/302 320/162	(i) Puffer fish: *F. niphobles* [26,166], *T. nigroviridis* [26], *T. biocellatus* [26], *Lagocephalus sceleratus* [29,47,48], *L. Lunaris* [27], *L. Spadiceus* [27], *Fugu poecilonotus* [59], *Fugu obscurus* [87], *Fugu pardalis* [24,26,125], *Fugu rubripes* [82], *Fugu niphobles* [26], *Takifugu oblongus* [138], *Takifugu vermicularis* [23], *Takifugu rubripes* [90], *Arothron immaculatus* and *Arothron nigropunctatus* [148,165], *Arothron firmamentum* [167], *A. reticularis* [27], *Chelonodon patoca* [23], *Xenopterus naritus* [27], *Tetraodon Nigroviridis* [26,27], *Tetradon biocellatus* [26], *Sphoeroides annulatus* [168], *S. Lobatus* [168], *S. lispus* [168], *Arothron meleagris* [168], *Canthigaster punctatissima* [168], *Takifugu niphobles* [169], *Pseudocaligus fugu* [169] and *Taeniacanthus* sp. [169] (ii) Gastropod: *Nassarius* spp. [25,28], *Gibbula umbilicalis* [47], *Monodonta lineata* [47], *Charonia lampas* [45,47,48], *N. nitidus* [25], *N. semiplicatus* [28], *N. papillosus* [12], *Niotha clathrata* [4,13,41,42], *Charonia sauliae* [46], *Nassarius (Alectricon) glans* [10,46], *Babylonia japonica* [46], *Zeuxis scalaris* [42,46], *Zeuxis samiplicutus* [5], *Z. sufflatus* [4,46], *Z. siquijorensis* [46], *Polinices didyma* [8], *Natica lineata* [8], *Oliva miniacea* [7], *O. mustelina* [7] and *O. nirasei* [7]. (iii) Crab: *Demania cultripes* [139,170], *Xanthias lividus* [67,171], *Carcinoscorpius rotundicauda* [63,172], *Demania toxica* [139], *Demania reynaudi* [65,139], *Lophozozymus incises* [139], *Lophozozymus pictor* [139], *Atergatopsis germaini* [139], *Atergatis floridus* [62,65,139], *Zosimus aeneus* [171], *Actaeodes tomentosus* [171] and *Camposcia retusa* [171]. (iv) Newt: *Taricha* spp. [36], *Taricha granulosa* [32,49,51,54,58], *Cynops ensicauda popei* [59], *Taricha torosa* [55,56], *Triturus* spp. [35], *Notophthalmus viridescens* [32,36,37,50,53,57,60], *Cynops* spp. [32], *Cynops pyrrhogaster* [52], *9Cynops ensicauda* [31,173], *Triturus alpestris* [35], *Triturus cristatus* [35], *Triturus helveticus* [35] and *Triturus vulgaris* [35] (v) Frog: *Brachycephalus ephippium* [33,34,72], *B. pernix* [72], *Polypedates* sp. [71] and *Atelopid* frogs [30] (vi) Blue-ringed octopus: *Hapalochlaena lunulata* [76,77,78], *Hapalochlaena fasciata* [76,78] and *Octopus maculosus* [94] (vii) Starfish: *Astropecten scoparius* [75] (viii) Sea slug: *Pleurobranchea maculata* [73,74] (ix) Copepod: *Pseudocaligus fugu* ectoparasitic on the panther puffer *Takifugu pardalis* [85] (x) Ribbon worm: *Cephalothrix* sp. (Nemertean) [22] and *Cephalothrix linearis* (Nemertean) [79] (xi) Bacteria: *Bacillus* spp. W-3 from *Fugu obscurus* [87], *Vibrio* spp. from xanthid crab *Atergatis floridus* [61], *Vibrio alginolyticus* from the starfish *Astropecten polyacanthus* [174], *Aeromonas* from *Takifugu obscurus* [88], *Bacillus* sp. from *Fugu obscurus* [87], *Bacillus* spp. and *Actinomycete* spp. from *Fugu rubripes* [82], *Microbacterium arabino galactanolyticum* [81], *Serratia marcescens* [81], *Vibrio alginolyticus* [81] from *Chelonodon patoca*, *Bacillus*, *Micrococcus*, *Alcaligenes*, *Caulobacter* and *Flavobacterium* from fresh water sediment [95], *Bacillus*, *Micrococcus*, *Acinetobacter*, *Aeromonas*, *Alcaligenes*, *Alteromonas*, *Flavobacterium*, *Moraxella*, *Pseudomonas* and *Vibrio* from deep sea sediment [21], *Alteromonas*, *Bacillus*, *Pseudomonas* and *Vibrio* spp. from the blue-ringed octopus, *Octopus maculosus* [94], *Roseobacter* sp. from the copepod *Pseudocaligus fugu*, parasite of puffer *Takifugu pardalis* [85], Copepods, *Pseudocaligus fugu* and *Taeniacanthus* sp., parasites of puffer *Takifugu niphobles* [169], *Aeromonas* from puffer fish, *Takifugu obscures* [88], and *Vibrio* Strain, LM-1 from the puffer, *Fugu vermicularis radiatus* [91]
7	4-epiTTX	319.101567	C_11_H_17_N_3_O_8_	320/302 320/162	(i) Puffer Fish: *F. niphobles* [26,166], *Takifugu nigroviridis* [26], *Fugu poecilonotos* [59], *Fugu pardalis* [24,125], *Takifugu oblongus* [138], *Tetraodon nigroviridis*, *Tetradon biocellatus* and *Lagocephalus sceleratus* [29,47], *Sphoeroides annulatus*, *S. lispus*, *Arothron meleagris*, *Canthigaster punctatissima* and *Pseudocaligus Fugu* [169] and *A. meleagris* [168] (ii) Gastropod: *Nassarius* spp. [28], *Nassarius glans* [10], *Charonia lampas* and *M. lineata* [47] and *N. semiplicatus* [86] (iii) Crab: *Demania cultripes* [170] (iv) Newt: *Cynops pyrrhogaster* [52], *Cynops ensicauda popei* [59], *Cynops ensicauda* [173], *Notophthalmus viridescens* [50,53,57], *Triturus* spp. [35] and *Taricha granulosa* [51] (v) Frog: *Brachycephalus ephippium* [33,34] (vi) Blue-ringed octopus: *Octopus maculosus* [94] (vii) Copepods: *Pseudocaligus Fugu* and *Taeniacanthus* sp., parasites of puffer *Takifugu niphobles* [169] (viii) Ribbon worm: *Cephalothrix* spp. [22] (ix) Bacteria: *Vibrio* Strain, LM-1 from the puffer, *Fugu vermicularis radiatus* [91]
8	6-epiTTX	319.101567	C_11_H_17_N_3_O_8_	320/302 320/162	(i) Puffer Fish: *Fugu poecilonotos*, (<LOD) [59] (ii) Newt: *Taricha* spp. [36], *Cynops ensicauda* [173], *Cynops ensicauda popei* [59], *Notophthalmus viridescens* [32,36,37,50,53,57,60], *Cynops pyrrhogaster* [52], *Triturus alpestris* [35], *Triturus cristatus* [35], *Triturus helveticus* [35], *Triturus vulgaris* [35], *Taricha granulosa* [32] and *Cynops* spp. [32] (iii) Frog: *Brachycephalus ephippium* [34,72]*, B. nodoterga* [72] and *B. pernix* [72]
9	Tetrodonic acid	319.101567	C_11_H_17_N_3_O_8_	NR	(i) Puffer Fish [127] (ii) Newt: *Cynops ensicauda* [173] (iii) Frog: *Brachycephalus ephippium* [33,34]
10	11-norTTX-6,6-diol	305.085917	C_10_H_15_N_3_O_8_	NR	Synthetic analogue [112]
11	5-deoxyTTX	303.106652	C_11_H_17_N_3_O_7_	304/286 304/176	(i) Puffer Fish: *Lagocephalus sceleratus* [29], *F. niphobles* [26], *T. nigroviridis* [26], *T. biocellatus* [26], puffer Fish [127], *Fugu poecilonotus* [59] and *Fugu pardalis* [24,125] (ii) Newt: *Cynops ensicauda popei* (<LOD) [59] (iii) Frog: *Brachycephalus ephippium, B. nodoterga* and *B. pernix* [72]
12	11-deoxyTTX	303.106652	C_11_H_17_N_3_O_7_	304/286 304/176	(i) Puffer Fish: *F. niphobles* [26], *Takifugu oblongus* [138], *T. nigroviridis* [26], *T. biocellatus* [26], *Lagocephalus sceleratus* [29], *Fugu poecilonotos* [59] and *Fugu pardalis* [24,125] (ii) Newt: *Cynops ensicauda* [59,173], *Cynops* spp. [32], *Taricha granulosa* [32] and *Notophthalmus viridescens* [32] (iii) Frog: *Brachycephalus ephippium, B. nodoterga* and *B. pernix* [72]
13	1-hydroxy-5,11-dideoxyTTX	303.106652	C_11_H_17_N_3_O_7_	NR	Newt: *Taricha granulosa* [59]
14	4,9-anhydro TTX	301.091002	C_11_H_15_N_3_O_7_	302/256 302/162	(i) Puffer Fish: *F. niphobles* [26,166], *T. nigroviridis* [26], *T. biocellatus* [26], *Fugu poecilonotos* [59], puffer Fish [127], *Fugu pardalis* [24,125], *A. meleagris* [168], *Lagocephalus sceleratus* [29,47], *Sphoeroides annulatus* [168] and *S. lispus,* [168] (ii) Gastropod: *Charonia lampas* [47] (iii) Xanthid crab: *Demania cultripes* [170] (iv) Newt: *Cynops ensicauda popei* [59], *Cynops pyrrhogaster* [52], *Notophthalmus viridescens* [50,53,57], *Triturus* spp. [35] and *Taricha granulosa* [51] (v) Frog: *Brachycephalus ephippium* [34]
15	6-epi-4,9-anhydroTTX	301.091002	C_11_H_15_N_3_O_7_	302/256 302/162	Newt: *Cynops pyrrhogaster* [52], *Notophthalmus viridescens* 53,57 and *Triturus* spp 35,175
16	AnhydroTTX	300.083177	C_11_H_14_N_3_O_7_^−^	302/256 302/162	(i) Puffer Fish: *Takifugu niphobles* [169], *Takifugu oblongus* [138] and *Pseudocaligus Fugu* [169] (ii) Gastropod: *Charonia lampas* [47], *Nassarius* spp. [28], *Zeuxis samiplicutus* and *Nassarius glans* [10], *Natica lineata* [8] and *Polinices didyma* [8] (iii) Crab: *Xanthias lividus* [67,139] and *D. Cultripes* [139] (iv) Newt: *Cynops ensicauda* [173] (v) Frog: *Brachycephalus ephippium* [33] (vi) Blue-ringed octopus: *Octopus maculosus* [94] (vii) Copepods: *Pseudocaligus Fugu* and *Taeniacanthus* sp., parasites of puffer *Takifugu niphobles* [169] (viii) Ribbon worm: *Cephalothrix* spp. [22] (ix) Bacteria: *Roseobacter* sp. from the copepod *Pseudocaligus Fugu*, parasite of puffer *Takifugu pardalis* [85] and *Vibrio* Strain, LM-1 from the puffer, *Fugu vermicularis radiatus* [91]
17	11-norTTX-6(S)-ol	289.091002	C_10_H_15_N_3_O_7_	290/272 290/162	(i) Puffer Fish: *Lagocephalus sceleratus* [29,47], *Fugu poecilonotos* [59], puffer Fish [127], *Fugu pardalis* [125] and *A. meleagris* [168] (ii) Frog: *Brachycephalus ephippium* [33] (iii) Newt: *Cynops ensicauda popei* (<LOD) [59] and *Arothron nigropunctatus* [148] (iv) Sea slug: *Pleurobranchaea maculata* [73]
18	11-norTTX-6(R)-ol	289.091002	C_10_H_15_N_3_O_7_	290/272 290/162	(i) Puffer Fish: *Lagocephalus sceleratus* [29,47], puffer Fish [127] and *Fugu niphobles* [147] (ii) Crab: *Atergatis Floridus* [62] (iii) Newt: *Cynops ensicauda* [173] (iv) Sea slug: *Pleurobranchaea maculata* [73]
19	1-hydroxy-8-epi-5,6,11-trideoxy TTX	287.111737	C_11_H_17_N_3_O_6_	288/162	(i) Puffer Fish: *Fugu poecilonotos* (<LOD) [59] (ii) Newt: *Cynops ensicauda popei* [59]
20	6,11dideoxyTTX	287.111737	C_11_H_17_N_3_O_6_	288/224	(i) Puffer Fish: *F. niphobles* [26], *T. nigroviridis* [26], *T. biocellatus* [26], *Fugu niphobles* and *Fugu poecilonotos* [59] and *Fugu pardalis* [125] (ii) Newt: *Cynops ensicauda popei* (<LOD) [59]
21	8,11dideoxyTTX	287.111737	C_11_H_17_N_3_O_6_	NR	Synthetic analogue [114,119]
22	5,6,11-trideoxy TTX	271.116822	C_11_H_17_N_3_O_5_	272/254 272/162	(i) Puffer Fish: *F. niphobles* [26], *Takifugu oblongus* [138], *T. nigroviridis* [26], *T. biocellatus* [26], *Lagocephalus sceleratus* [29,47], puffer Fish [127] *Fugu poecilonotos* [59] and *Fugu pardalis* [24,125] (ii) Gastropod: *Nassarius* spp. [28] and *Charonia lampas* [47]
23	8-epi-5,6,11-trideoxy TTX	271.116822	C_11_H_17_N_3_O_5_	272/162	Newt: *Cynops ensicauda popei* [59]
24	4-epi-5,6,11-trideoxyTTX	271.116822	C_11_H_17_N_3_O_5_	NR	Puffer fish [127]
25	1-hydroxy-4,4a-anhydro-8-epi-5,6,11-trideoxyTTX	269.101172	C_11_H_15_N_3_O_5_	270/162	(i) Puffer Fish: *Fugu poecilonotus* (<LOD) [59] (ii) Newt: *Cynops ensicauda popei* [59]
26	4,9-anhydro-5,6,11-trideoxy TTX	253.106257	C_11_H_15_N_3_O_4_	NR	Puffer fish [59]
27	4,9-anhydro-8-epi-5,6,11-trideoxy TTX	253.106257	C_11_H_15_N_3_O_4_	254/162	Newt: *Cynops ensicauda popei* [59]
28	4,4a-anhydro-5,6,11-trideoxy TTX	253.106257	C_11_H_15_N_3_O_4_	NR	Puffer fish [59]
29	4-epi-11-deoxyTTX	NR	NR	NR	Newt (*Cynops ensicauda*) [173]
30	4,9-anhydro-11-deoxyTTX	NR	NR	NR	Newt (*Cynops ensicauda*) [173]

Note: NR, Not Reported.

## 3. Chemical Synthesis of TTX and Its Analogues

**Figure 2:**
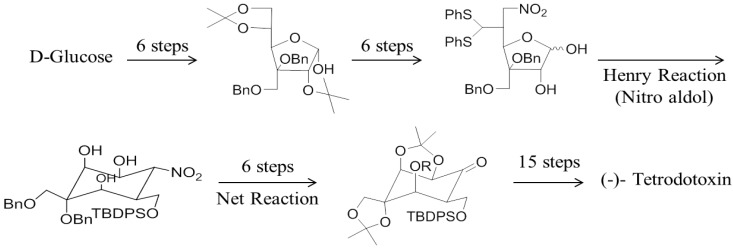
Chemical synthesis of TTX from glucose [119] (*R* = MOM).

Several groups have synthesised TTX (Figure 2) and some of its analogues including 8,11-dideoxyTTX and 5,11-dideoxyTTX using glucose as precursor molecule [113,114,115,116,117,118,119,120]. The key intermediate compound for the synthesis of TTX can be obtained either from quinone [113] or carbohydrate [119]. Umezawa *et al*., 2010 [177] have synthesized ^13^C-labelled 5,6,11-trideoxytetrodotoxin and ^13^C-labelled 4-epi-5,6,11-trideoxytetrodotoxin from aldehyde. Adachi *et al*., 2013 [120] have synthesized (-)-5,11-dideoxytetrodotoxin from the enone. Sato *et al*., 2012 [119] have done stereo selective synthesis of optically active TTX from d-Glucose. Nishikawa *et al*., 2003 have done stereo controlled synthesis of 11-deoxyTTX and 8,11-dideoxytetrodotoxin from levoglucosenone [116]. Ohyabu *et al*., 2003 [117] achieved the asymmetric synthesis of tetrodotoxin from 2-acetoxy-tri-*O*-acetyl-d-glucal. 

However in general, chemical synthesis of TTX involves many complex steps (average no. = 23–67) which generate low yields (0.34%–1.82%) of the target compound [111]. Additionally, in most synthesis it is necessary to develop methods for purification. These complications make most laboratory synthesis approaches for TTX and its analogues unfeasible for commercial scale-up.

## 4. Aetiology of TTX

### 4.1. Biosynthesis of TTX

**Figure 3:**
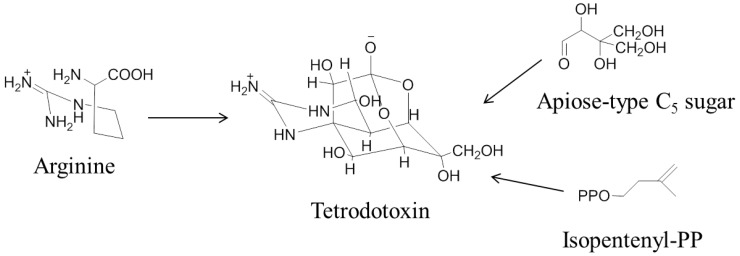
Proposed biosynthesis of tetrodotoxin from arginine [49,111].

The biosynthetic origin of TTX *in vivo* has yet to be agreed. It is supposed that arginine is the precursor moiety for TTX production within the organism (Figure 3) [49]. 

### 4.2. Aetiology of TTX among Marine and Fresh Water Organisms

**Figure 4:**
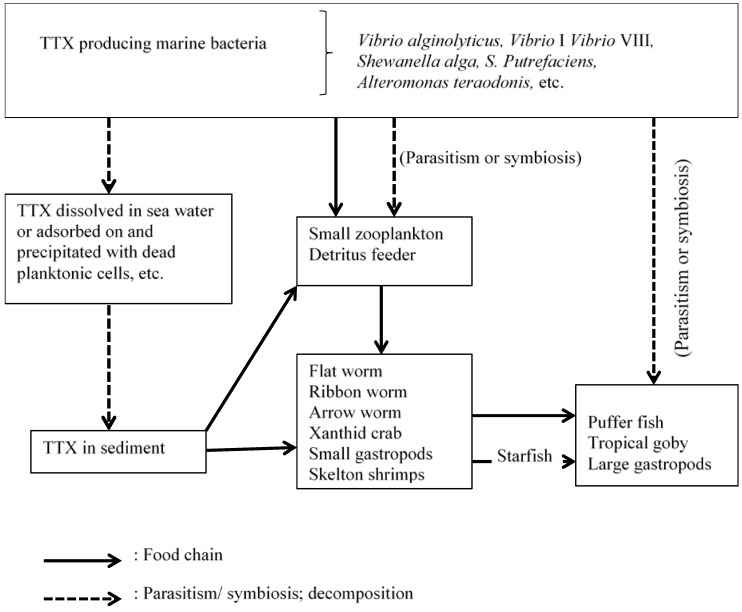
Proposed mechanism of TTX accumulation in marine animals [14].

The ecology of marine organisms [178] and terrestrial organisms [179] with respect to TTX have been discussed before. There have been several theories proposed for the formation and bio-transfer of TTX. TTX is believed to bio-accumulate via the marine food chain [178] (Figure 4).

It is known that several species of bacteria and other microorganisms often live within larger marine animals in a supposed or in an established mutually advantageous symbiosis [180,181,182]. The following species of bacteria are known TTX producers and have been isolated from various marine organisms: *Vibrio alginolyticus* from starfish, *Astropecten polyacanthus* [174]; *Vibrio* spp. from the puffer fish, *Fugu vermicularis radiates* [91]; *Aeromonas* from puffer fish, *Takifugu obscures* [88]; *Vibrio* and *Pseudomonas* spp. from gastropod, *Niotha Clathrata* [80]. Chau *et al*., 2011 [111] have provided a comprehensive account of the distribution of TTX producing bacteria in many organisms.

A small gastropod, *U. suturale* had been isolated from the digestive gland of starfish, *A. scoparius* is a known progenitor of TTX and anhydroTTX [75]. This suggests an exogenous (external) origin for TTX in this starfish.

An exogenous origin for TTX is also suspected for certain toxic crabs which feed on small gastropods known to contain TTX and on marine sediments containing TTX-positive bacteria (food vectors include: *Vibrio* VIII from xanthid crab [61]; *Vibrio* and *Pseudomonas* spp. from gastropod, *Niotha Clathrata* [80]; *Bacillus*, *Micrococcus*, *Aeromonas*, *Alteromonas*, *Moraxella*, *Vibrio* and *Actinetobacter* spp. from deep sea sediment [21] and, *Bacillus*, *Micrococcus*, *Alcaligenes*, *Caulobacter* and *Flavobacterium* spp. from fresh water sediment [95]). Therefore it is assumed that crabs obtain TTX from the food chain.

The assumption of an exogenous origin of TTX in fresh water and marine organisms is also supported by other studies. It has been shown that puffer fish, *Takifugu rubripes* [90] and grey side-gilled sea slugs, *Pleurobranchaea maculata* [74] become non-toxic when they are fed on a TTX-free diet. Also when puffer fish, *Takifugu rubripes* were fed with a TTX-containing diet their toxicity increased significantly [14]. 

Contrarily, Matsumura, 1998 [183] have demonstrated that TTX levels in the embryos of puffer fish, *Takifugu niphobles* increase until hatching; emphasizing its endogenous (internal) origin. Other evidence for an endogenous origin for TTX in gastropods is that TTX was higher in the muscle than in the digestive gland of the snail *N. lineata* [184]. In addition, TTX toxicity in the edible gastropod, *Polinices didyma* was higher in the muscle tissue than in the digestive gland [184]. Of course in the case of the gastropods, it is possible that after initial ingestion of TTX, the toxin could have then migrated and remained in tissue compartments outside of the digestive region.

### 4.3. Aetiology of TTX among Terrestrial Animals

In newts the levels of TTX and 6-epiTTX are higher in the skin rather than in the liver [51]. The hypothesis here is that TTX is biosynthetically produced by the creature as a defense mechanism to deter and immobilise predators. However, researchers have found that the newt, *N. viridescens* [37] becomes non-toxic when it is fed a TTX-free diet suggesting, that at least in this case that TTX has an exogenous origin.

Pires Jr. *et al*., 2005 [72] studied TTX in frogs belonging to the *Brachycephalus* family, noting that the degree of colouration of the frog can be related to its toxicity. They have found the highest TTX levels in the skin followed by liver and ovaries of frogs belonging to the genus *B. ephippium* and *B. pernix* which are bright yellow. However *B. nodoterga*, which has cryptic colouration, was found to be non-toxic. Interestingly, the infamous “poison dart” frogs belonging to *Dendrobatidae* family are brightly coloured and are well known for high toxicity, though their toxicity derives not only from TTX. The bright colouration protects these frogs from predators who instinctively avoid highly coloured prey [185]. This strongly suggests toxic frogs naturally synthesise the toxin; however studies have not been conducted to definitively corroborate this. 

Toxicity in two snails, on which crabs feed, *Polinices didyma* and *Natica lineata* collected from Southern Taiwan were higher (1563 and 2590 MU/specimen respectively) than that of these species collected from Western Taiwan (8–123 and 5–95 MU/specimen respectively) [8]. These studies indicate that there is regional variation in toxicity that subsequently correlates with the toxicity of crabs from these regions. This would imply an exogenous origin of TTX in both these snails and the crabs higher up the food chain.

Despite all of these assumptions, the exact origin and pathway for the synthesis and bio-transfer of TTX is not yet fully known and requires further investigation.

## 5. Biochemistry

TTX is a sodium channel blocker. Binding of TTX to voltage gated sodium channel results from the interaction between the positively charged guanidine group on the TTX with the negatively charged carboxylate groups on the side chains in the mouth of the sodium channel [98,186,187]. TTX binding prevents diffusion of sodium ions through the sodium channels. This in turn prevents depolarization and propagation of action potentials in nerve cells leading to the loss of sensation. Biotransformation of TTX inside the human or mammalian body is yet to be widely investigated. Some information is available on transformation of TTX and/or its analogues inside the bodies of puffer fish [134] and grey side-gilled sea slugs, *Pleurobranchaea maculata* [74]. Wood *et al*., 2012 [74] have shown that degradation/transfer of TTX in the heart tissue is the fastest, while TTX degrades/transfers relatively slowly from the gonads. Additionally, there was a very high level of TTX in eggs. In the puffer fish body, TTX enters the liver first after ingestion. Then it is transferred mainly to the skin in the male and the reproductive organs in the female [103,134]. As discussed in Section 6, TTX binding proteins isolated from marine puffer fish can have a major role in the biotransformation of TTX in the puffer fish body [188,189]. 

Whether the analogues of TTX are biosynthesized or formed as metabolites or act as precursors for the synthesis of TTX in affected bearing organisms is yet to be verified. 

## 6. Resistance to TTX in TTX Bearing Organisms

Many researchers have been intrigued as to how TTX bearing organisms are themselves resistant to the toxic effects of TTX. The reason is because in these animals the aromatic amino acid chain in the p-loop region of domain I in the sodium channels is replaced by a non-aromatic amino acid and this prevents the sodium channels in these species from being blocked [103,190,191].

Resistance to TTX can also be related to the presence of a TTX-binding protein present in the shore crab, *Hemigrapsus sanguineus* [192], in puffer fish, *Takifugu niphobles* [188] and *Fugu pardalis* [189,193] and also in five gastropod species: *Polinices didyma*, *Natica lineata*, *Olivaminiacea*, *O. Mustelina* and *O. hirasei* [194].

## 7. Clinical Study

There are four grades of TTX poisoning described by Fukuda and Tani, 1941 [195]. 

Grade 1: perioral numbness and paresthesia (sensation of tingling, tickling, prickling, pricking, or burning of a person’s skin), with or without gastrointestinal symptoms. 

Grade 2: lingual numbness (numbness of face and other areas), early motor paralysis and incoordination, slurred speech with normal reflexes.

Grade 3: generalized flaccid paralysis (reduced muscle tone without other obvious cause), respiratory failure, aphonia (the inability to produce voice due to disruption of the recurrent laryngeal nerve), and fixed/dilated pupils (conscious patient).

Grade 4: severe respiratory failure and hypoxia (inadequacy of oxygen), hypotension (low blood pressure), bradycardia (resting heart rate of under 60 beats per minute), cardiac dysrhythmias (irregular heartbeat) and unconsciousness may occur. 

As mentioned previously, the grade of TTX poisoning depends upon the amount of TTX ingested, the time after ingestion of TTX, the hydration state of body and the general health status of the victim prior to intoxication [105]. 

### 7.1. Time after Ingestion of TTX

During the Bangladesh outbreak of TTX poisoning in 2008 [19], the onset of symptoms was observed within 30 min of ingestion of puffer fish in 66% of the total number of cases, within 31–60 min in 24% of cases, within 61–90 min in 7% of cases and within 91–120 min in 2% of cases. The poisoning symptoms decreased gradually over 8–28 h after ingestion of the contaminated puffer fish with no residual side effects. 

During the TTX poisoning outbreak in Israel between 2005 and 2008, the onset of symptoms was seen within 10–60 min after ingestion. Whereas during the TTX poisoning event in Taiwan in April 2001 [6], the onset of symptoms was typically within 6 h of ingestion, with a delay of up to 20 h in a few cases. The victims became well without any remaining side effects and were discharged 48–72 h after ingestion.

TTX can be found in blood within less than 24 h after its ingestion. But it can be found in urine after 4 days from the time of ingestion [100,196]. Therefore, it is important to collect urine and blood samples from affected patients within 24 h after ingestion for clinical diagnosis. 

### 7.2. Amount of TTX Ingested

The victims of the Bangladesh outbreaks in April 2008 [19] ingested less than 50–200 g of puffer fish. The victims who died had ingested more than 100 g of TTX contaminated puffer fish. 

During the Bangladesh outbreak in April 2002 [9], 37 victims were affected by TTX poisoning. There was not any correlation between the quantity of puffer fish ingested and the number of deaths. Surprisingly, 4 deaths were seen among 10 victims who had ingested just 51–100 g of fish but only 3 deaths were seen among those that had ingested larger portions (101–500 g) of the fish.

### 7.3. Physical Status of Victim

There was no significant correlation between urine TTX levels and blood TTX levels and the severity of the poisoning symptoms found during the Bangladesh outbreak [19]. This is because urine TTX levels depend not only on the amount of TTX ingested but also on the hydration state of the victim. While analysing TTX in urine, it is necessary to do a creatinine adjustment in order to eliminate the effects of the variations in the urinary outputs between patients [135]. Zimmer, 2010 [97] found a strong correlation between blood TTX levels and the poisoning symptoms. This could be due to similar hydration states of the victims.

During the TTX poisoning episode that occurred in Taiwan in April 2001 [106], urine and blood samples of 4 of the victims were collected about 10 h after ingestion and analysed by LC-MS. The blood TTX level of one victim who died was 40.6 nM (12.96 ng/mL) and that of the survivors’ varied between 4.5 and 28.6 nM (1.44–9.13 ng/mL). The urine TTX level of the victim who died was 325 nM (103.71 ng/mL). The urine TTX level of the two survivors was lower (190 nM (60.63 ng/mL) and 47 nM (15 ng/mL)). However a higher level of TTX was found in the urine of one of the survivors (344 nM (109.77 ng/mL)) which was greater than the patient who died (325 nM (103.71 ng/mL)) and both of them had ingested almost the same quantity of fish (100 and 90 g respectively). This suggests that the victim who died might not have excreted the TTX fast enough resulting in higher levels in the blood [40.6 nM (12.96 ng/mL)] which led to death just 4 h after ingestion. All of the survivors recovered well without long term effects. This shows individual variability of patient outcomes with regard to TTX poisoning.

### 7.4. Health Status of Victim

TTX poisoning was observed in a wide range of age groups in the TTX outbreak in Bangladesh [19]. Therefore, all age groups are susceptible to intoxication by TTX toxin. TTX poisoning in people suffering from diabetic neuropathy (nerve damage), uremia (illness accompanying kidney failure) and Na-K-adenosine-triphosphate deficiency modify the symptoms of TTX poisoning [196]. Diabetic neuropathy is a neurological disorder related to diabetes mellitus. It affects all peripheral nerves including the pain fibres, motor neurons and the autonomic nervous system; therefore it can affect all organs. TTX poisoning in a patient with diabetic neuropathy can lead to severe synergistic effects. During the TTX poisoning incident in Taiwan in April 2001 [6], one of the victims who died from TTX poisoning had diabetic neuropathy. This victim ingested 80 g of puffer fish which was less than the amount ingested by one of the survivors (100 g) without any residual effects. 

Na-K-adenosine-triphosphatase (EC 3.6.3.9) [197], also known as the sodium-potassium pump is an anti-porter enzyme present in the plasma membrane of all animal cells. The Na^+^/K^+^-ATPase enzyme pumps sodium out of cells, while pumping potassium into cells. TTX poisoning in persons suffering from Na-K-adenosine-triphosphate deficiency can have severe effects.

Uremic syndrome (uremia) [198] is a serious complication of chronic kidney disease and acute renal failure. An excess of urea and waste products accumulate in the body of uremic patients due to kidney dysfunction. This can led to a range of symptoms which are similar to TTX poisoning symptoms including; nausea, vomiting, changes in mental status such as confusion, reduced awareness, agitation, psychosis, seizures and coma, abnormal bleeding such as bleeding spontaneously or profusely following a minor injury, heart problems such as an irregular heartbeat, inflammation and an excess of fluid in the sac that surrounds the heart and increased pressure on the heart. Nakashima *et al*., 2007 [199] reported mild TTX poisoning in a uremic patient (a 52-year-old woman) from Japan, who had numbness of the lips and weakness in the legs after ingestion of puffer fish. While her symptoms were not severe and she did not require respiratory support, her condition did not improve after 2 days of hospitalization. Haemodialysis (extracorporeal removal of waste products such as creatinine and urea and free water from the blood when the kidneys are in a state of renal failure) was therefore performed on hospital days 3, 4 and 5, and resulted in a marked improvement of symptoms. As a consequence of uremia, TTX accumulated in her body due to its delayed excretion. This suggests that haemodialysis might be helpful for the treatment of TTX poisoning [199].

### 7.5. Clinical Findings

Clinical manifestations of TTX poisoning had been reported during various outbreaks [6,9,19,105,106]. Kanchanapongkul *et al*., 2008 studied 280 cases of TTX poisoning caused by ingestion of eggs of the horseshoe crab, *Carcinoscorpius rotundicauda*, between 1994 and 2006 that were admitted to Chon Buri Hospital in Thailand [172]. Out of 245 medical records; 100 were in stage 1; 74 were in stage 2; 3 were in stage 3 and 68 were in stage 4 of intoxication. 

During the Bangladesh outbreak in April 2008 [19], the blood TTX level was >9 ng/mL (28.20 nM) in 7 of the victims who died (5 within 15–30 min and 2 after 4 h of ingestion of puffer fish). Only 3 victims survived, in which the blood TTX level was between 9.3 and 10 ng/mL (29.14–31.34 nM). This suggests that a blood TTX level of 9 ng/mL (28.20 nM) or greater may be considered as potentially lethal for human beings. 

In the Bangladesh outbreak [19], routine haematological, biochemical and ECG tests were normal in all of the victims. In the Israel outbreak [105], these tests were also normal in almost all of the victims, however one victim had hypercapnia (too much CO_2_), one had hypokalemia (low level of potassium in blood which is maintained by the Na^+^/K^+^ pump) and two had elevated levels of CPK (creatine phosphokinase) which is a marker enzyme for myocardial infarction (heart attack), rhabdomyolysis (severe muscle breakdown), muscular dystrophy, autoimmune myositides (chronic inflammation of striated muscle) and acute renal failure. In this outbreak [105], one of the victims was likely to have had a seizure (a neurological disorder; often associated with a sudden and involuntary contraction of a group of muscles and loss of consciousness). Previously it had been reported that seizures were correlated with TTX poisoning [38]. 

### 7.6. Treatment

There is presently no antidote available for TTX poisoning. Chew *et al*., 1984 [200] have tried to make use of an anti-cholinesterase drug for treating TTX. During the large TTX incident in Bangladesh [19], 21 victims were given anticholinesterases; neostigmine and atropine but it wasn’t seen to improve their condition. This is because anticholinesterases reverse the blocking action at neuromuscular junction at the motor end plates only. Whereas TTX blocks sodium channels of motor neurons and muscle membranes. 

One of the severely poisoned victims of the TTX outbreak in Israel [105], had been administered 0.4 mg of naloxone intravenously which showed an equivocal response. Naloxone has a high affinity for μ-opioid receptors in the central nervous system and is used for treating depression of the central nervous system and respiratory system. 

Currently, the only treatment for TTX poisoning is to provide the victim with respiratory support until the TTX is excreted completely. Endotracheal intubation can be provided to facilitate ventilation of the lungs. Mechanical ventilation may also be provided. During the TTX poisoning outbreak in Israel [105], patients were given respiratory support and recovered within 4 days. During the TTX poisoning event in Thailand [172], victims were treated with endotracheal intubation and mechanical ventilation. Out of 245 victims, 239 patients (97.5%) completely recovered, 5 patients (2%) died, and 1 patient (0.4%) suffered anoxic brain damage.

In the case of early stage TTX poisoning victims are given activated charcoal in order to help the adsorption of TTX to prevent its absorption through the stomach [201]. Gastric lavage (the passage of a tube via the mouth or nose down into the stomach followed by sequential administration and removal of small volumes of liquid) can be performed in TTX poisoning in order to reduce its severity. This procedure should be performed within 60 min after ingestion of TTX [202]. In the Bangladesh outbreak in April 2002 [9], 37 patients were given gastric lavage and neostigmine treatment along with respiratory support. But there are some risks associated with gastric lavage treatment such as laryngospasm (involuntary muscular contraction of the laryngeal cords), hypoxia (inadequacy of oxygen), bradycardia (a resting heart rate of under 60 beats per minute), epistaxis (nosebleed), hyponatremia (reduced levels of sodium in the blood), hypochloremia (reduced levels of chloride ions in blood), water intoxication or mechanical injury to the stomach. 

Intravenous fluids are also given in order to maintain fluid-electrolyte balance in the body during TTX poisoning. An antiemetic is given which is effective against vomiting and nausea. As mentioned in paragraph 7.4, haemodialysis might also be useful for the treatment of TTX poisoning [199].

Antibodies against TTX have been used successfully *in vivo* [203,204]. Xu *et al*., 2005 synthesized antibody against TTX [130]. This antibody was able to neutralise the toxic effect of TTX both *in vitro* and *in vivo*. A monoclonal antibody for TTX (anti-TTX) is available commercially from Hawaii Biotech, Inc., Aiea, HI, USA [121,136]. However, studies on the efficacy of this monoclonal antibody *in vivo* have not been published [121], but this may herald the advent of a new type of approach to the treatment of TTX poisoning in the future.

### 7.7. Application of TTX in the Medical Field

Some researchers are trying to make use of the analgesic activity of TTX to treat various types of pains such as severe cancer pain [205,206,207]. A low dose of TTX has also been shown to help in reducing cue-induced increases in heroin craving and associated anxiety [208]. 

## 8. The Distribution of TTX and Its Analogues

### 8.1. Geographic Distribution of TTX

#### 8.1.1. Geographic Distribution of TTX in Freshwater and Marine Organisms

TTX poisoning is very common in Japan, Taiwan, Bangladesh and Southeast Asia (Table 1). Most commonly it is associated with the consumption of puffer fish and sometimes by the ingestion of gastropods or crabs. In the last decade, TTX was found in puffer fish [29,48,131] and gastropod [44,45,48] collected from European countries as well. A number of researchers have formulated the theory of “Lessepsian migration” for explaining the new occurrence of TTX in European regions. In 1869, the opening of Suez canal caused migration of many Red Sea species through the new waterway which settled in the Eastern Mediterranean e.g., occurrence of *Lagocephalus sceleratus* in the Mediterranean Sea. Hence, this phenomenon is called “Lessepsian migration” [105,131,209]. The occurrence of several migrant marine species have been reported from Mediterranean Sea such as *Lagocephalus sceleratus*, *Mobulamobular*, *Tylosuruschoram*, *Scarusghobban* and *Tetrapturus belone* [210,211,212,213,214,215,216]. Ballast water can also cause the transfer of TTX containing organisms from Asian waters to European waters. Over the last 20 years, spreading of marine mucilage in the Mediterranean Sea was observed due to sea surface warming [99]. This helps the survival of migrated species in Mediterranean Sea.

Some cases of TTX poisoning caused by the consumption of puffer fishes have been observed in the US also [2,108,110]. But those species of fish were imported from either Japan or from other places. TTX had been found in puffer fishes, *Sphoeroides annulatus*, *S. lobatus*, *S. lispus*, *Arothron meleagris* and *Canthigaster punctatissima* collected from the coast of the Baja in the California Peninsula, Mexico [168]. Mexico is the second largest puffer fish exporter in the world. 

Occasionally natural calamities can also cause small changes in the marine ecology of a region and thus the occurrence of new and/or displaced species. Chulanetra *et al*., 2011 [27] state that the ecology of marine organisms including puffer fish might have been affected by the Tsunami in the Andaman Seas which originated from the earthquake in the Indian Oceans in 2004. They studied the toxicity of 155 puffer fishes caught off the Gulf of Siam and from the Andaman Sea near Thailand, during April to July 2010. Among 125 puffers from the Gulf of Siam, 18 were *Lagocephalus lunaris* and 107 were *L. spadiceus* which were found previously in 2000–2001 in the same region. They also reported the presence of TTX in *L. spadiceus* for first time. In 1992–1993, nine marine puffer fish species were caught in the Andaman Seas, *Arothron immaculatus*, *A. stellatus*, *Chelonodon patoca*, *Diodon hystrix*, *Lagocephalus lunaris*, *L. inermis*, *L. spadiceus*, *L. sceleratus*, and *Xenopterus naritus*. Out of these, five puffers, *A. immaculatus*, *C. patoca*, *L. lunari*, *L. sceleratus* and *X. Naritus* were found to be positive for TTX [27]. In 2011, thirty puffers were collected from the Andaman Sea: 28 *Tetraodon nigroviridis* and two juvenile *Arothron reticularis*; the two new species totally replaced the nine species found previously in the same region during the 1992–1993 study and some of these were found positive for TTX [27]. 

#### 8.1.2. Geographic Distribution of TTX in Terrestrial Animals

TTX was found in newts from the USA, Japan, Germany and Italy; *Notophthalmus viridescens* from Canada and USA [37,51,53,54,55,57,58], *Notophthalmus viridescens and Taricha* from North America [36], *Cynops ensicauda popei* from Okinawa, Japan [59], *Cynops pyrrhogaster* from Japan [52], *Triturus* spp. from Germany [35] and *Triturus alpestris* from Italy [32].

TTX was also found in frogs belonging to the Brachycephalidae family from the Brazilian Atlantic rain forest [33,72], from the Central American frog *Atelopus chiriquiensis* [30] and in the Rhacophoridid frog, *Polypedates* sp. from Bangladesh [71]. 

### 8.2. Organism Specific Distribution of TTX and Its Analogues

The occurrence of 26 natural analogues of TTX had been reported to-date (Figure 1). The name tetrodotoxin is based on the name of the order of animals called tetradontiformes first suggested by Dr. Yoshizumi Tahara in 1909. He isolated TTX from the ovaries of globefish, *Fugu* [20]. Up to 1964, TTX was believed to be present only in puffer fish. In 1964 Mosher [217] found TTX in the California newt, *Taricha torosa*. Subsequently, it was also found in frogs, shell fish, star fish, ribbon worms, sea slugs and bacteria (Table 3). 

#### 8.2.1. Distribution of TTX and Its Analogues in Puffer Fish

TTX is found in marine, fresh water and brackish water organisms [93]. The presence of TTX in puffer fish is well documented. Table 3 gives details of puffer fish species containing TTX and its analogues. Puffer fish of the Tetradontidae family are toxic while puffer fish from the *Diodontodae* and *Ostracitidae* families are usually non-toxic [93]. *Lagocephalus wheeleri*, *L. gloveri* and *Takifugu xanthipterus* are considered as non-toxic species of puffer fish [93,218]. Nakashima *et al*., 2007 [199] reported mild TTX poisoning caused by *Lagecephalus wheeleri* in Japan for the first time. Simon *et al*., 2009 [219] also found TTX in *Lagecephalus wheeleri* from Malaysian waters identified by LC (liquid chromatography) but it was not lethal to mouse in the mouse bioassay. Nagashima *et al*., 2001 [218] have shown that non-toxic *Takifugu xanthipterus* contains less toxic TTX derivatives. This indicates that TTX might be converted into less toxic derivatives in these species. *T. nigroviridis* was more toxic than *Lagocephalus lunaris*, *L. Spadiceus* and *Arothron reticularis* collected from the Andaman seas [27].

Female puffer fish are more toxic than male puffer fish as they accumulate TTX in the ovaries and eggs during the spawning period [46]. Jang *et al*., 2006 [24] studied distribution of TTX analogues in *Fugu pardalis*. 5,6,11-trideoxyTTX (especially in ovaries) was the major and 5-deoxyTTX and 11-deoxyTTX were minor TTX analogues in all tissues. Whereas 4,9-anhydroTTX was the major analogue in liver, 4-*S*-CysteinylTTX was detected in liver, spleen, gall, and intestine in 1–6 mole percentage of the total of all the TTX analogues.

Vázquez *et al*., 2000 [168] found that 4,9-anhydroTTX was the major and 4-epiTTX was minor analogue of TTX in puffer fishes, *Arothron meleagris* (black phase), *A. meleagris* (golden phase), *Sphoeroides annulatus* and *S*. *lispus*. 4,9-anhydroTTX was not found in both *S*. *lobatus* and *Canthigaster punctatissima* whereas, 4-epiTTX was found in very low concentration in *Canthigaster punctatissima.* In general, the level of TTX was higher than the level of 4,9-anhydroTTX in liver. While the level of 4,9-anhydroTTX was higher than the level of TTX in mucus. Diener *et al*., 2007 [138] found that TTX was the major analogue in liver, muscle and skin whereas trideoxyTTX was the major analogue in ovaries of *Takifugu oblongus*. Jang *et al*., 2010 [26] found that TTX and 5,6,11-trideoxyTTX were the major TTX analogues whereas 4-epiTTX 4,9-anhydroTTX, 5-deoxyTTX and 11-deoxyTTX were minor TTX analogues in *Fugu niphobles*, *Tetraodon nigroviridis* and *Tetradon biocellatus*. 6,11-dideoxyTTX was the major analogue in almost all tissues of *F. niphobles*, but it was the minor analogue in *Tetraodon nigroviridis* and *Tetradon biocellatus*. Kudo *et al*., 2012 [59] found that the levels of TTX, 5,6,11-trideoxyTTX and anhydro-5,6,11-trideoxyTTX were highest among all the analogues of TTX. Whereas, the level of 4,9-anhydroTTX was highest among all the remaining minor analogues of TTX; 4-epiTTX, 5-deoxyTTX, 11-deoxyTTX, 6,11-dideoxyTTX and 11-norTTX-6(*S*)-ol in *Fugu poecilonotus*. Rodríguez *et al*., 2012 [29] found that 5,6,11-trideoxyTTX was the major TTX analogues, followed by 11-deoxyTTX, 11-norTTX-6(*S*)-ol, and TTX. While 4-epiTTX, 4,9-anhydroTTX, 5-deoxyTTX and 11-norTTX-6(*R*)-ol were minor analogues of TTX in *Lagocephalus sceleratus*.

#### 8.2.2. Distribution of TTX and Its Analogues in Gastropod

TTX and some of its analogues were found in gastropod species: *Gibbula umbilicalis-* monodeoxy (TTX (0.063 µg/g)); *Monodonta lineata* (TTX (0.090 µg/g) and 4-epiTTX (0.021 µg/g)) and *C. lampas* (5,6,11-trideoxyTTX (0.006 µg/g)) [47]; *C. lampas* (TTX (315 µg/g) and 5,6,11-trideoxyTTX (1004 µg/g)) [45]; *N. nitidus* (TTX (1350 µg/g)) [25]; *N. semiplicatus* (TTX (26.1 µg/g) and 4-epiTTX (3.37 µg/g)) [86] and *N. papillosu* (TTX (42–60 µg/g)) [12]. TTX and its analogues trideoxyTTX, 4-epiTTX, anhydroTTX and oxoTTX were detected in the nassariid species [28]. Usually, the levels of TTX in gastropod are lower than those in puffer fishes. But gastropods are also capable of inducing TTX poisoning [4,5,7,8,10,12,42,45,46]. In general TTX and 5,6,11-trideoxyTTX are the major while 4-epiTTX is the minor analogue of TTX found in gastropods.

#### 8.2.3. Distribution of TTX in Sea Slug, Star Fish, Blue-Ringed Octopus, Ribbon Worm and Bacteria

TTX had been occasionally found in the sea slug, star fish and ribbon worm. TTX was detected in sea slugs, *Pleurobranchaea maculata* which caused poisoning in dogs on the beaches of Hauraki Gulf, Auckland, New Zealand [73]. The presence of TTX in sea slugs, *Pleurobranchaea maculata* have also been reported by Wood *et al*., 2012 [74]. Along with TTX, low levels of 11-norTTX have been found in *Pleurobranchaea maculata* by McNabb *et al*., 2010 [73].

TTX has been found in a starfish species, *Astropecten scoparius* [75], a ribbon worm, *Cephalothrix linearis* (Nemertean) [22,79] and blue-ringed octopuses, *Hapalochlaena fasciata* and *Hapalochlaena lunulata* [76] (Table 3). TTX, 4epi-TTX and anhydroTTX were found in ribbon worm, *Cephalothrix* sp. present on the shells of cultured oysters [22]. No other analogue of TTX has been reported in either starfish, or blue-ringed octopus todate.

TTX and anhydroTTX were found in bacteria, *Shewanella woodyi* and *Roseobacter* sp. isolated from the copepod *Pseudocaligus fugu* parasitic on the panther puffer *Takifugu pardalis* [85]. TTX, 4-epi-TTX, and anhydroTTX were found in *Vibrio* Strain, LM-1, from the puffer fish *Fugu vermicularis radiatus* [91]. 

#### 8.2.4. Distribution of TTX and Its Analogues in Terrestrial Animals

As previously stated, TTX can be found in several species of newts: *Notophthalmus* [32,36,37,50,53,57], *Cynops* [31,32,52,59] and *Triturus* spp. [35], crabs: *Demania cultripes*, *Demania toxica*, *D. reynaudi*, *Lophozozymus incisus*, *L. Pictor* and *A. germaini* [62,139,170] and in frogs and toads: *Atelopus* [70,143] and *Brachycephalus* [33,34,72]. 

Along with TTX, other related analogues were also found in newt and salamander: TTX [32,51,54], 6-epiTTX [32,36], 11-oxoTTX [36], 4-epiTTX [51], 4,9-anhydroTTX [51] and 11-deoxyTTX [32] in *Taricha* spp*.* Recently four new analogues, 8-epi-5,6,11-trideoxyTTX (MW 271.2710), 4,9-anhydro-8-epi-5,6,11-trideoxyTTX (MW 253.2557), 1-hydroxy-8-epi-5,6,11-trideoxyTTX (MW 287.2704) and 1-hydroxy-4,4a-anhydro-8-epi-5,6,11-trideoxyTTX (MW 269.2551) have been isolated from newt which are not present in puffer fish [59]. 

Pires Jr. *et al*., 2002 [33] found TTX, 4-epiTTX, 4, 9-anhydroTTX, 11-norTTX-(*S*)-ol and tetrodonic acid in the frog, *Brachycephalus ephippium*. Pires Jr. *et al*., 2003 [34] identified 11-oxoTTX also from the same species of frog. Pires Jr. *et al*., 2005 [72] have studied the toxicity of three species of frogs: *Brachycephalus ephippium*, *B. nodoterga* and *B. Pernix* belonging to the Anuran family, Brachycephalidae. Toxicity was highest in the skin followed by the liver and ovary in *Brachycephalus ephippium* and *B. prenix.* 11-oxoTTX, TTX, 4-epiTTX, 4,9-anhydroTTX, 5-deoxyTTX and tetrodonic acid were found in *Brachycephalus ephippium* while TTX, 4,9-anhydroTTX, 5-deoxyTTX and tetrodonic acid were found in *B. Prenix* by LC-FLD (liquid chromatography-fluorescent detection)*.* Traces of 11-oxoTTX, TTX, 4-epiTTX, 4,9-anhydroTTX, 5-deoxyTTX and tetrodonic acid were found in *B. nodoterga* using LC-FLD but *B. nodoterga* extract was non-toxic by mouse bioassay. Also, two unknown compounds having mass spectral signals at *m*/*z* 330 and 348 which may be attributed to TTX analogues but could not be unequivocally identified. The 11-oxo TTX analogue is commonly found in frogs but rarely seen in puffer fish and newts. 

TTX and anhydroTTX were found in xanthid crab, *Xanthias lividus* [67]. Along with TTX small amounts of anhydroTTX were also found in crab, *D. cultripes* [139]. TTX was the major while 4-epiTTX and 4,9-anhydroTTX were minor analogues of TTX in xanthid crab, *Demania cultripes* [170]. 11-oxotetrodotoxin and 11-nortetrodotoxin-6(R)-ol have been reported in a xanthid crab, *Atergatis floridus* [62].

6-epi TTX is more commonly found in terrestrial organisms than in marine or in fresh water species. Table 3 gives details of the occurrence of TTX and its analogues in a variety of organisms.

### 8.3. Tissue Specific Distribution of TTX and Its Analogues in All Organisms

**Puffer fish:** TTX is found in all tissues of puffer fish. The levels of TTX vary among species of puffer fish. A study of the tissue distribution of analogues of TTX in *Fugu Pardalis* was compiled by Jang and Yotsu-Yamashita in 2006 [24]. They found that the liver and ovary are more highly toxic than the muscle and testis in wild puffer fish. 4-Cys TTX was found in the liver, spleen, gall bladder and intestine of *Fugu Pardalis* but not in the ovary [24]. 

In marine puffer fish, the liver tissue is toxic throughout the year except during the spawning season but the ovaries become more toxic perhaps to protect the organism from predators. In puffer fish that inhabit brackish water and freshwater regions toxicity is higher in the skin than in marine species [93].

The average level of 4-epiTTX in puffer fish is ca. 10% (mol/mol) of TTX [128] and 5,6,11-trideoxy TTX was the major analogue in all tissues of the puffer fish, *Fugu pardalis* [125]. The levels of TTX analogues were highest in the ovary of puffer fish *Takifugu oblongus* than in any of the other tissue compartments. Among all the analogues, the levels of 5,6,11-trideoxyTTX was the highest and was located in the ovaries [138]. 

**Gastropod:** TTX is mainly found in the muscles of gastropods. Some amounts of TTX are also found in the digestive glands. TTX and its analogues trideoxyTTX, 4-epiTTX, anhydroTTX and oxoTTX were detected in the gastro pod nassariid [28]. TrideoxyTTX was the major toxin in all the samples [28]. The toxicity of TTX was higher in the muscle tissue than in the digestive gland in *N. lineata* [184]. The toxicity of edible portions of *Polinices didyma* and of *Natica lineate* was higher than that in the digestive gland [8]. 

**Newt:** TTX is found mainly in the skin of newt. In newt (*Taricha granulosa*), TTX levels were high in the skin of adult newt and in the yolk of embryo. TTX levels decrease during the development of larvae and larvae are almost non-toxic [58]. 

**Crab:** Less information is available regarding tissue specific distribution of TTX and its analogues in crab. TTX has been found in xanthid crabs, *Demania cultripes*, *Demania toxica*, *D. reynaudi*, *Lophozozymus incises*, *L. Pictor* and *A. germaini* in southern Taiwan. Toxicity was higher in the viscera (4.0–11.9 MU/g) than in the appendages and cephalothorax in all of the species tested. Toxicity of the appendages was 1.6–12.3 MU/g while toxicity of cephalothorax was 1.5–3.6 MU/g among all the species [139]. 

**Frog:** Skin is the main toxic tissue in frog. Pires Jr. *et al*., 2005 [72] have studied the toxicity of three species of frogs; *Brachycephalus ephippium*, *B. nodoterga* and *B. pernix* belonging to the Anuran family, Brachycephalidae. Toxicity was highest in the skin followed by the liver and ovaries in *Brachycephalus ephippium* and *B. prenix* which was studied by mouse bioassay. They have not given the toxin profile of individual tissues. Chiriquitoxin (CqTX) was found in the skin and in the eggs of *Atelopus chiriquiensis* [30,70].

**Other organisms:** TTX was found in the skin of the gobies [220] and in the head of arrow worms [221] and in the proboscis of the ribbon worm [79]. Blue-ringed octopuses contain TTX in the posterior salivary gland, the skin and eggs. Williams and Caldwell, 2009 [76] studied 14 tissue types from blue-ringed octopuses. In their study TTX was found in the posterior salivary gland (PSG), arm, mantle, anterior salivary gland, digestive gland, testes contents, brachial heart, nephridia, gill and oviducal gland of *Hapalochlaena fasciata*. But in *H. lunulata* TTX was found only in the PSG, mantle and ink. 

### 8.4. TTX Co-occurrence with Other Marine Toxins

Occurrence of either minor amounts of paralytic shellfish poisons (PSP) along with TTX, or major amounts of PSP along with traces of TTX have been reported in many species of puffer fish, crabs and gastropods (*N. Clathrata*, *Polinices didyma* and *N. Lineata*) [8]. Sometimes species that are susceptible to TTX intoxication have been found to be free of TTX (or have TTX only at very low levels) while they have been found to have other toxins such as PSP present at the time of testing. Hydroxysaxitoxin has been found in the xanthid crab, *Demania cultripes* from the Philippines [170]. Saxitoxin, decarbamoyl saxitoxin have been confirmed in Cambodian freshwater puffer fish, *Tetraodon turgidus* (which is not resistant to TTX) [222]. The presence of GTX-2 and GTX-3 has been noted in *Colomesusasellus*, an Amazonian (Brazil) freshwater puffer fish [223]. Saxitoxin and decarbamoyl saxitoxin have been found in *Fugu pardalis* [24]. Saxitoxin and decarbamoyl saxitoxin have been identified in the marine puffer *Arothron firmamentum* [167]. GTX-1,2,3 and 4 have been found in the Taiwanese crab, *Xanthias lividus* [67]. The puffers *Sphoeroide snephelus*, *S. testudineus*, and *S. Spengleri* have tested positive for saxitoxin from the Indian River Lagoon, Florida [224]. Gonyautoxin 2, gonyautoxin 3 and saxitoxin (STX) and neoSTX, decarbamoylSTX and STX have been found in the xanthid crab *Zosimus aeneus* [66]. Saxitoxin, decarbamoyl saxitoxin, gonyautoxins 2 and 3, decarbamoyl gonyautoxins 2 and 3 have been found in the freshwater puffer fish, *Tetraodon cutcutia* and *Chelonodon patoca* from Bangladesh [225]. Gonyautoxin 1-4 in the xanthid crab, *Atergatis floridus* and gonyautoxin 2-4 and neosaxitoxin in the xanthid crab, *Demania reynaudi* from Taiwan [65], saxitoxin, neosaxitoxin and decarbamoyl saxitoxin in the freshwater puffers, *Tetraodon leiurus* and *Tetraodon suvatii* from Thailand [226], gonyautoxin-3; GTX3, GTX2, and saxitoxin in the gastropod *Rapana venosa* from Japan [227], saxitoxin in the freshwater puffer, *Tetraodon fangi* from Thailand [228], and saxitoxin in the southern (*Sphoeroide snephelus*), checkered (*Sphoeroide stestudineus*), and band tail (*Sphoeroide spengleri*) puffer fish from the US [229] were found. TTX was found to co-occur in small quantities with PSP in *A. Germaini* in northern Taiwan. TTX and GTX were also found to co-occur in *D. reynaudi and L. pictor* in northern Taiwan [139]. TTX was found in samples together with palytoxin in *Demania cultripes, D. reynaudi and L. pictor* of Philippine and in *L. pictor* from Singapore [139]. 

**Figure 5:**
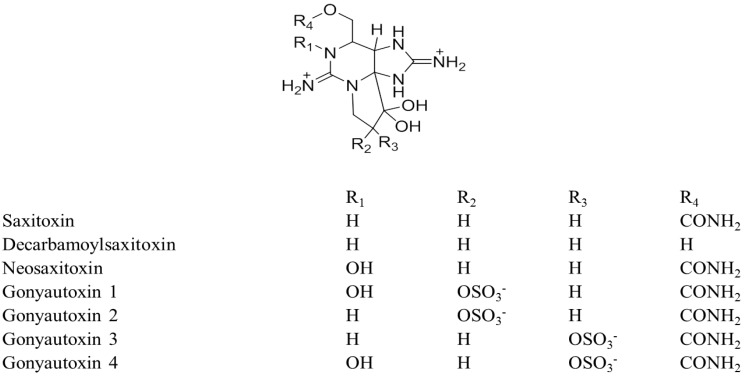
Structures of paralytic shellfish poisons (PSP) toxins.

Both, TTX and PSPs (Saxitoxin, neosaxitoxin, decarbamoyl saxitoxin, hydroxysaxitoxin, gonyautoxin 1, 2, 3 and 4) are neurotoxins which block the sodium channels. The structures of TTX (Figure 1) and STX (Figure 5) are similar; they bind to a common site, which is present at the external mouth of the sodium channels [230]. Symptoms of paralytic shellfish poisoning are similar to TTX poisoning [201]. So there is a risk of misdiagnosis in such poisoning events. Several treatments for TTX poisoning have been discussed earlier in the text (Section 7.6). Interestingly, 4-aminopyridine was observed to reverse the effect of saxitoxin and tetrodotoxin in mice without any side effects such as seizure or convulsions [231,232]. The optimal 4-AP dose was determined as 2 mg/kg (im) [232]. 

## 9. Chemical Stability and Toxicity of Analogues

TTX is heat stable and water soluble [233]. It is an aminoperhydroquinazolone. 4-epiTTX and 4,9-anhydroTTX are in chemical equilibrium with TTX while deoxy analogues (5-deoxy TTX, 11-deoxy TTX, 6,11-dideoxy TTX and 5,6,11-trideoxyTTX), 11-oxo TTX and 4-S-cysteinyl TTX are not in chemical equilibrium with TTX [26,89,166]. 

The toxicity of analogue is based on the number and position of hydroxyl groups present in the structure. Yotsu-Yamashita *et al*., 1999 [112] have studied the effects of specific modifications of a number of hydroxyls of TTX on its affinity to rat brain membrane. The results showed that hydroxyls at C-6 and C-11 play an important role in the binding of these toxins to the sodium channels, acting as hydrogen bond donors. In an earlier study, Yang and Kao showed that hydroxyls at C-4, C-6, C-9, C-10 and C-11 also play role in binding to the sodium channel [70]. 

11-OxoTTX is 4 to 5 times more toxic than TTX [34]. The additional OH group of 11-oxoTTX probably binds to the sodium channels with hydrogen bonding more efficiently than TTX. 11-oxo TTX is rare in marine animals and it is found predominantly in frogs.

5-Deoxy TTX, trideoxy TTX, 4-Cys TTX and anhydro TTX have negligible toxicity. The toxicity of 5,6,11-trideoxyTTX is less than that of TTX as it has less hydroxyl groups compared with that of TTX. Fewer hydroxyl groups serve to reduce the binding affinity of 5, 6, 11-trideoxyTTX to the sodium channels. Jang and Yotsu-Yamashita, 2007 [125] have found that the toxicity of 6,11-dideoxyTTX was higher than a synthetic analogue 8,11-dideoxyTTX.

Jang and Yotsu-Yamashita, 2007 [24] make the assumption that 4-CysTTX might be the metabolite of 4,9-anhydroTTX formed by the reduction of glutathione as they have found high levels of 4,9-anhydroTTX and 4-CysTTX in liver, spleen, gall and intestine but not in the ovaries of selected species. 

Kudo *et al*., 2012 [59] hypothesized that TTX is the oxidation product of 5,6,11-trideoxyTTX in the TTX-producing marine organisms.

Kono *et al*., 2008 [166] and Wang *et al*., 2011 [134] state that TTX is metabolised in the puffer fish, *Fugu niphobles* and thus produces different analogues; most abundantly 4,9-anhydroTTX. In puffer fish TTX accumulates in the liver and then slowly transfers to the skin.

## 10. Miscellaneous Studies

Saoudi *et al*., 2007 [15] showed that in the cooked flesh together with the water of cooking, the toxic effect was more pronounced than in the raw flesh. Shiu *et al*., 2003 found that the level of TTX in cooked gastropod, *Polinices didyma* was in the range of 30–261 MU/specimen [8], while Taylor *et al*., 2011 found TTX levels in the range of 374–655 µg/100 g in the cooked flesh and in the soup broth [136]. This indicates that cooking does not remove or degrade TTX. Anraku *et al*., 2013 [234] have found that the traditional salting and fermentation process does reduce TTX levels in the ovaries of puffer fish, *Takifugu stictonotus*.

## 11. Historical Perspective on Analytical Methods Used for TTX and Its Analogues

### 11.1. Bioassays

Receptor binding assay [235]; immunological methods (e.g., ELISA) [23,52,88,121,134,236,237,238] and mouse bioassay [27,28,81,92,131,223,239] have all been used for TTX analysis. The mouse bioassay is the method that is most frequently applied. All of the above methods can be used successfully to identify TTX but not all of its analogues. Researchers tried to improve the accuracy and repetition of bioassays. Recently Stokes *et al*., 2012 [240] have developed a competitive inhibition enzymatic immunoassay method which has high accuracy and repeatability at concentration range of 10–100 ng/mL. However, this method doesn’t identify any of the other analogues of TTX and also it involves the use of expensive antibodies (primary and secondary). There are also ethical issues regarding the use of live animal bioassays (the mouse bioassay) when more accurate and precise analytical methods that can both qualitatively and quantitatively profile TTX and its analogues are widely available.

### 11.2. Chemical Assays

Surface plasma resonance [136], electrophysiological assays [97,98,191,232]; IR [96]; NMR [53,59,96,125,173,241,242]; GC-MS [7,8,85,137,139,169,170,171]; LC-FLD and LC-MS (Table 4, Table 5, Table 6, Table 7, Table 8 and Table 9) have been developed and used for the determination of TTX by many researchers. In addition NMR, GC-MS, LC-FLD and LC-MS provide the benefit of finding not only TTX but related analogues along with co-occurring toxins in samples thus allowing a more comprehensive risk assessment of produce intended for human consumption.

Yotsu-Yamashita *et al*., 1999 have quantified 6-epiTTX, 11-deoxyTTX and 5,6,11-trideoxyTTX by ^1^H-NMR using TTX standard [112]. The difficulty with NMR is that in real samples intense interference from matrix components can compromise the quality of the spectra.

Due to huge variations in fluorescence intensities of different members of the TTX family as well as interfering background signals from the matrix, LC-FLD is not a good choice for the routine analysis of real samples. The fluorescence intensities of 6-epiTTX and 11-norTTX-6(*R*)-ol are 20-fold and 10-fold higher respectively than that of TTX while the fluorescence intensities of 5-deoxyTTX and 11-deoxyTTX are 1/20 and 1/100 lower than that of TTX [72,149].

GC-MS has been used to screen for the presence of TTX. However because TTX is non-volatile, it needs to be converted into its volatile derivative before analysis by GC-MS. This is a disadvantage for the method as derivatisation requires a large amount of sample, and the method suffers from poor reproducibility and is also time consuming. 

Therefore LC-MS and especially LC-MS/MS are generally regarded as the best choice for the determination of TTX and related compounds (Table 4, Table 5, Table 6, Table 7, Table 8 and Table 9).

### 11.3. Historical Overview of LC-MS/MS Methods for TTX and Its Analogues

Table 4, Table 5, Table 6, Table 7, Table 8 and Table 9 provide a brief summary of the LC-MS methods that have been used for the analysis of TTX and its analogues in a variety of sample types: puffer fish (Table 4), trumpet shell/ gastropod, sea slug and octopus (Table 5), newt (Table 6), crab and frog (Table 7), bacteria (Table 8) and Human blood/ urine (Table 9).

#### 11.3.1. Extraction and Clean Up Methodologies

Table 10 summarises the extraction processes and percentage (%) recoveries of TTX from different matrices. The % recovery varies from 80% to 90% for most of the extraction protocols when applied to real samples. However, the recovery of TTX was only 50% for newt samples [35]. 

**Table 4:** LC-MS methods for TTX and its analogues from puffer fish.

Species	Extraction	Column	Mobile phase	Method	Analyte *	LOD and LOQ	Linear Range	Reference
*Lagocephalus sceleratus* (Gmelin, 1789) (Liver, GI-tract, gonad (ovary/testis), muscle and skin)	0.1% AA	Zorbax 300SB-C_3_ Sunfire C_18_ XBridge™ Amide	Isocratic: 1% ACN + 10mM TMA + 10 mM AF, pH 4 (For Zorbax 300SB-C_3_) A: 1% ACN + 20 mM AHB + 20 mM Am-OH + 10 mM AF, pH 4 and B: 5% ACN + 20 mM AHB + 20 mM Am-OH + 10 mM AF, pH 4 (For Sunfire C_18_) A: 10 mM AF + 10 mM FA in H_2_O B: 5 mM AF + 2 mM FA in ACN:H_2_O, 95:5 (For XBridge™ Amide)	LC-MS/MS and CID-MS/MS	6, 7, 11, 12, 14, 17, 18, 22	LOD:16 ng/mL at S/N > 3 LOQ:63 ng/mL at S/N > 10	62.5–2000 ng/mL	[29]
*Lagocephalus sceleratus* (Muscle)	ASE and SE (0.03 M AA)	Acquity UPLC BEH HILIC	A: 5% ACN B: 95% ACN + 1% AA pH 3.5	LC-M/MS	6	For Solvent Std LOD: 0.074 ng/mL LOQ: 0.123 ng/mL For Matrix-matched Std LOD: 7.3 µg/kg and LOQ: 24.5 µg/kg at S/N = 3 and 10	5–500 ng/mL (Solvent Std) 50–3000 µg/kg (Matrix-matched Std)	[48]
Potka or Tepa fish (Cooked fishAnd blood and urine of victim)	1% AA + 80% MeOH	C_30_ UG-5	A: 30 mM AHB, pH 5 in H_2_O B: 10 mM AF, pH 5 in 1% ACN	LC-FLD	6, 7, 14	NR	NR	[19]
*Lagocephalus lunaris*, *L. spadiceus*, *Tetradon nigroviridis* and *Arothron reticularis* (Reproductive tissue, digestive tissue, liver, muscle and skin)	0.1% AA, ethyl acetate Defat *, CharAd ^†^	ZIC-HILIC	A: 10 mM AF + 10 mM FA in H_2_O B: 5 mM AF + 2 mM FA in 80% ACN	LC-MS/MS	6, 11/12, 16	NR	NR	[27]
Puffer fish (ovary)	0.05 M AA, ODS-SPE, Ultra filtration (0.22 µ)	Atlantis HILIC Silia	10 mM AF, pH 3.5 + ACN (22:78, v/v)	LC-MS (SIR) LC-MS/MS (CID)	6, 7, 9, 11, 14, 17/ 18, 20, 22	SIR mode LOD: 0.1 ng/mL at S/N = 3 LOQ: 0.25 ng/mL at S/N = 10	0.25–100 ng/mL	[127]
*Takifugu rubripes* and *Takifugu niphobles* (Muscle, skin, liver, gonad)	1% AA	Puresil C_18_	30 mM HFB + 1 mM Am-acetate, pH 5.0	LC-MS	6, 7, 14	NR	NR	[167]
*Fugu niphobles* (Ovary/testis, liver, intestine, dorsal skin and dorsal muscle) *Tetraodon nigroviridis* and *Tetraodon biocellatus* (Whole body)	0.05 M AA, C_18_-SPE, CHCl_3_ Defat *, CharAd ^†^	TSKgel Amide-80	16 mM AF, pH 5.5 in ACN (3:7, v/v)	LC-MS/MS	6, 7, 11, 12, 14, 20, 22	NR	NR	[26]
*Lagocephalus* sp. (Cooked fish)	1% AA, CHCl_3_ Defat *	TSK-GEL Amide-80	5 mM AF + 26.5 mM FA in ACN:H_2_O, 70:30	LC-MS	6	NR	NR	[108]
*Tetraodon turgidus* and *Tetraodon* sp. (Skin, muscle, liver, intestine, gonad; ovary/testis)	NM	RP-18	1 mM TBA-PO_4_, pH 5.8	HPLC-FLD	PSP toxins (STX, neoSTX, GTX1–4, dcSTX, dcGTX2 and 3)	NR	NR	[222,243]
*Fugu poecilonotus* (Liver) *Fugu niphobles* (Whole body)	Sephadex G-10 Gel filtration (For *F. poecilonotus*) 0.1% AA, 50% CharAd ^†^ (For *Fugu niphobles*)	For *F. poecilonotus*: ODS-5; LC-FLD For * Fugu niphobles*: HILIC; LC-MS	5 mM AHB + 50 mM Am-acetate, pH 5 in 3% ACN; LC-FLD (For *F. poecilonotus*) 5 mM AHB + 50 mM Am-acetate, pH 5 in 3% ACN; LC-FLD (For * Fugu niphobles*)	LC-FLD LC-FLD and LC-MS	1, 4, 6, 7, 14 (on ODS-5) and 11, 12, 20, 22 (on HILIC)	NR	NR	[89,166]
*Fugu niphobles* (Liver, intestine, gonad, bone, muscle, skin, other organs; viscera)	0.1% AA	ODS-5	20 mM AHB, pH 5 + 10 mM Am-acetate, pH 5 in 3% MeCN	LC-FLD	6, 7, 14	NR	NR	[89]
*Takifugu oblongus* (Liver, gonad; ovary/testis, muscle, skin, other organs; viscera)	0.1% AA	SeQuant ZIC- HILIC	A: 10 mM AF + 10 mM FA in water B: 5 mM AF + 2 mM FA in 80:20 ACN:H_2_O	LC-MS/MS	6, 7, 12, 16, 22	LOD:0.09 ng (TTX), 0.14 ng(AnhydroTTX), 0.20 ng (11-deoxy TTX)	0.25–10 ng (TTX) 0.25-5.8 ng (AnhydroTTX) 0.20-5 ng (11-deoxy TTX)	[138]
*Fugu pardalis* (Ovary)	0.05 M AA, EtOAc Defat *, CharAd ^†^, Bio-Gel P2 and Hitachi gel 3011C filtration	TSK gel Amide-80	16 mM AF, pH 5.5 in ACN (3:7, v/v)	LC-MS	6, 7, 11, 12, 14, 17, 20, 22	NR	NR	[24,125]
*Fugu pardalis* (Ovary, testis, liver, spleen, gall, skin, intestine, kidney and muscle)	0.1% AA, Cosmosil 75 C_18_-OPN resin	C_30_ UG-5 (LC-FLD) TSK-GEL Amide-80 (LC/MS)	30 mM AHB + 10 mM AF in 1% ACN, pH 5 (C_30_ UG-5) 16 mM AF, pH 5.5 in ACN (3:7, v/v) (TSK-GEL Amide-80)	LC-FLD LC-MS	1, 6, 11, 12, 14, 22,	LOD 0.07 pmole (LC-MS)	NR	[24,149,175]
*Fugu poecilonotus* and *F. pardalis* (Ovary)	0.05 M AA, CharAd ^†^	C_30_ UG-5 (LC-MS/MS and LC-FLD)	20 mM AHB + 10 mM AF in 1% ACN, pH 4 (LC-MS/MS) 30 mM AHB + 10 mM AF in 1% ACN, pH 5 (LC-LFD)	LC-MS/MS LC-LFD	6, 7, 10, 11, 14, 17, 18, 22	LOD 0.7 pmol at S/N 2	50–1000 pmol	[149]
*Takifugu xanthopterus* (Liver)	0.05 M Tris-Ac, pH 8.2; Sephacryl S-400 column filtration, 0.03 M AA, DCM Defat *, Bio-Gel P-2 filtration	YMC AM OSD	[218]	LC-FLD	6, 7, 9, 11	NR	NR	[218]

Note: (Analyte*) Refer to Table 3 for the name of the TTX analytes.

**Table 5:** LC-MS methods for TTX and its analogues from trumpet shell, gastropods and octopus.

Species	Extraction	Column	Mobile phase	MS	Analyte *	LOD and LOQ	Linear Range	Reference
**Trumpet shell**								
*Charonia lampas* (Viscera and muscle)	ASE ^‡^ and SE ^‡‡^ (0.03 M AA)	Acquity UPLC BEH HILIC	A: 5% ACN B: 95% ACN + 1% AA pH 3.5	LC-MS/MS	6	For Solvent Std LOD: 0.074 ng/mL LOQ: 0.123 ng/mL For Matrix-matched Std LOD: 7.3 µg/kg LOQ: 24.5 µg/kg at S/N = 3 and 10	5–500 ng/mL (Solvent Std) 50–3000 µg/kg (Matrix-matched Std)	[48]
*Charonia lampas lampas* (Digestive gland)	NM	NM	NM	LC-MS/MS	6, 22	NR	NR	[45]
**Gastropod**								
*Nassarius* spp.	[28]	[28]	[28]	HPLC-MSn (Ion trap) and HPLC-FLD	4, 6, 7, 16, 22	NR	NR	[28]
*Gibbula umbilicalis, Monodonta lineata* and *Charonia lampas*	1% AA, DCM Defat *, C_18_ SPE	XBridge™ Amide (LC- MS/MS) Waters Acquity UPLC BEH Amide (UPLC-MS/MS)	For both, A: 10 mM FA + 10 mM AF in H_2_O B: 2 mM FA + 5 mM AF in ACN: H_2_O, 95:5	LC-MS/MS UPLC-MS/MS	6, 7, 11/12, 14, 17/18, 22	For LC- MS/MS LOD: 16 ng/mL at S/N > 3 LOQ: 63 ng/mL S/N > 10 For UPLC-MS/MS LOD: 1.7 ng/mL at S/N > 3 LOQ: 5 ng/mL S/N > 10	50–2000 ng/mL (LC-MS/MS) 31.25–3000 ng/mL (UPLC-MS/MS)	[47]
Grey side-gilled sea slug, *Pleurobranchaea maculata* (Whole body)	50% MeOH, Strata Phenomonex SPE	TSK-GEL amide 80	A: 10% ACN + (90% 50 mM FA + 2 mM AF in H_2_O) B: 90% ACN + (10% 50 mM FA + 2 mM AF in H_2_O)	LC-MS/MS	4, 6, 12, 16, 17/18	LOD: 5 ng/mL (S/N = 50)	5–250 ng/mL	[73]
**Blue-ringed octopus**								
*Hapalochlaena fasciata* and *H. lunulata*	0.05 N AA	Synergi 4 µ Hydro-RP 80A C_18_	0.97% Heptafluorobutyric acid + 0.29% AA in 3% ACN (pH adjusted to 5.0 with NH_4_OH)	Q-TOF MS	6	NR	500 ng/mL to 0.5 mg/mL	[76]
Blue-ringed octopus (*Hapalochlaena fasiata* and *H. lunulata*) (Posterior salivary gland, arm, dorsal mantle, ventral mantle, anterior salivary gland, digestive gland, testes conts./egg/paralarva, oviducal gland, brachial heart, nephridia, gill)	0.05 N AA	Synergi 4 µ Hydro-RP 80A C_18_	3% ACN + 0.97% HFB + 0.29% AA, pH 5	LC-FLD	6	NR	500 ng/mL–0.5 mg/mL	[178]

Note: (Analyte*) Refer to Table 3 for the name of the TTX analytes.

**Table 6:** LC-MS methods for newts.

Species	Extraction	Column	Mobile phase	MS	Analytes *	LOD and LOQ	Linear Range	Reference
*Cynops ensicauda popei* (Whole body)	O.2 M AA, Hexane Defat *, CharAd ^†^, Bio-Rex 70 and Hitachi gel 3011C SPE	TSK gel G1000PW (HILIC)	16 mM AF, pH 5.5 + ACN (3:7, v/v)	LC-MS/MS	6, 7, 8, 11, 12, 14, 17, 19, 20, 23, 25, 27	NR	NR	[24,26,59,175]
*Notophthalmus viridescens* (Whole body)	0.1% AA + 70% EtOH, CharAd ^†^	Develosil C_30_ UG-5	1% ACN + 20 mM AHB + 10 mM AF, pH 4.0	LC-FLD	4, 6, 8	NR	NR	[37]
*Notophthalmus viridescens* (Whole body, liver and skin)	0.1% AA in 70% MeOH	Develosil C_30_ UG-5	1% ACN + 30 mM AHB + 10 mM AF, pH 5.0	LC-FLD	6, 7, 8, 14, 15,	LOD 0.4 pmol	50–1000 pmol	[57,149]
*Triturus* spp. (Whole body)	0.1% AA in 70% MeOH, CharAd ^†^	Develosil (C_30_-UG-5)	30 mM AHB + 10 mM AF, pH 5	LC-FLD	6, 8	LOD 100 ng/g (TTX) 40 ng/g (6-epi TTX)	NR	[35]
*Taricha granulosa* (Skin)	0.1 M AA	Synergi 4 µ Hydro-RP 80A	50 mM Am-acetate + 60 mM AHB, pH 5 in 1% ACN	LC-FLD	6	NR	NR	[54,176]
*Notophthalmus viridescens* (Whole body)	1% AA in 70% MeOH	Develosil C_30_-UG-5	1:11 vol.% ACN, 30 mM AHB + 10 mMAF, pH 5.0	LC-FLD	4, 6, 7, 8, 14, 15	NR	NR	[53]
*Taricha granulosa* (Skin)	0.1 M AA	Synergi 4 µ Hydro-RP 80A	50 mM Am-acetate + 60 mM AHB, pH 5 in 1% ACN	LC-FLD	6, 7, 14	NR	NR	[51]
*Cynopus ensicauda* (Skin)	0.05 M AA, CharAd ^†^	C_30_- UG-5 (LC-MS/MS and LC-FLD)	20 mM AHB + 10 mM AF in 1% ACN, pH 4 (LC-MS/MS) 30 mM AHB + 10 mM AF in 1% ACN, pH 5 (LC-LFD)	LC-MS/MS LC-LFD	6, 7, 8, 11, 12, 14, 15, 17, 18	LOD: 0.7 pmol at S/N 2	50–1000 pmol	[149]
*Notophthalmus viridescens* (Whole body)	1% AA in 70% MeOH	Develosil C_30_-UG-5 (HPLC-FLD and LC-MS)	30 mM AHB in 1% ACN, pH 5 (HPLC-FLD) 20 mM AHB + 10 mM AF in 1% ACN, pH 4 (LC-MS)	HPLC-FLD LC-MS	6, 7, 8, 11, 12, 14, 17, 18	NR	NR	[50]
*Cynops pyrrhogaster* (Whole body)	0.1% AA	Puresil C_18_	30 mM HFB + 1 mM Am-acetate, pH 5	LC-MS	6, 7, 8, 15	NR	NR	[52]

Note: (Analyte*) Refer to Table 3 for the name of the TTX analytes.

**Table 7:** LC-MS methods for TTX and its analogues from crab and frog.

Species	Extraction	Column	Mobile phase	MS	Analytes *	LOD and LOQ	Linear Range	Reference
**Crabs**								
*Demania cultripes*, *D. toxica*, *D. reynaudi*, *Lophozozymus incisus*, *L.pictor* and *Atergatopsis germaini* (Appendage, cephalo-thorax and viscera)	1%AA in MeOH, C_18_ cartridge	ODS-3	30 mM HFB + 1 mM Am-acetate, pH 5	LC-MS	6, 7, 16	LOD: 0.005 µg/mL	0.03–3 µg/mL	[52,139,]
*Xanthias lividus* (Appendage, cephalothorax and viscera)	1%AA in MeOH, DCM Defat *, Bio-Gel P-2 filtration	[67]	[67]	HPLC	6, 16	NR	NR	[67]
**Frogs**								
*Brachycephalus ephippium, B. nodoterga* and *B. pernix* (Whole Body, skin, liver and ovary)	MeOH:AA (96:4), Amberlite GC-50 SPE, CharAd^†^	CLC-ODS (LC-FLD) Puresil C_18_ (LC-MS/MS)	0.06N HFB + 0.001N Am-acetate, pH 5 (CLC-ODS) 30 mM HFB + 1 mM Am-acetate, pH 5 (Puresil C_18_)	LC-FLD LC-MS/MS	4, 6, 7, 8, 9, 11, 12, 14, 17	NR	NR	[33,52,72,176]
*Brachycephalus ephippium* (Skin)	1%AA in MeOH, Petroleum ether Defat*, CharAd^†^	CLC-ODS (LC-FLD) Puresil C_18_ (LC-MS/MS)	0.06N HFB + 0.001N Am-acetate, pH 5 (CLC-ODS) 30 mM HFB + 1 mM Am-acetate, pH 5 (Puresil C_18_)	LC-FLD LC-MS/MS	4, 6, 7, 8, 9, 14	NR	NR	[33,34,52,176]
*Polypedates* sp. (Skin, muscle and viscera)	80% EtOH, pH 2, DCM Defat*, CharAd^†^, 1% AA in 20% EtOH, Bio-Gel P2 and Bio-Rex 70 filtration	Inertsil ODS-3	60 mM (NH_4_)_3_PO_4_, pH 5 + 10 mM HSA in 2% ACN	LC-FLD	6, 8, 14, 15	NR	NR	[71]

Note: (Analyte*) Refer to Table 3 for the name of the TTX analytes.

**Table 8:** LC-MS methods for TTX and its analogues from bacteria.

Species	Extraction	Column	Mobile phase	MS	Analytes *	LOD and LOQ	Linear Range	Reference
*Aeromonas* strain from ovary of puffer fish, *Takifugu obscurus*	0.1% AA, CharAd ^†^, Bio-Gel P2 and C_18_ SPE	ACQUITY UPLC BEH HILIC	A: 0.2% FA in H_2_O B: 0.2% FA in ACN	Q-TOF MS	6	NR	0–250 ng/mL	[88]
*Shewanella woodyi* and *Rosebacter* sp. from copepod, *Pseudocaligus fugu*; ectoparasite of puffer fish, *Takifugu pardalis*	0.1% AA, C_18_ SPE, CharAd ^†^	[85,169]	Asakawa *et al*. 2003 and Ito *et al*. 2006	Asakawa *et al*. 2003 and Ito *et al*. 2006	6, 7, 16	NR	NR	[85,169]
Vibrio strain, LM-1 from the intestine of puffer fish, *Fugu vermicularis radiates*	DCM Defat *, 0.03 M AA, Bio-Gel P-2 filtration	YMC-pack AM-314 octyldecyl silane	0.05M HSA + 0.05M KH_2_PO_4_, pH 7 in MeOH	LC-FLD	6, 7, 16	NR	NR	[91]
*Nocardiopsis dassonvillei* from the ovary of puffer fish, *Fugu rubripes*	0.1% AA, CharAd ^†^, Bio-Gel P2 and Bio-Rex 70 filtration	Bio-Rex 70	MeOH	LC-MS	6	NR	NR	[82,92]

Note: (Analyte*) Refer to Table 3 for the name of the TTX analytes.

**Table 9:** LC-MS methods for TTX and its analogues from human urine and blood.

Sample	Extraction	Column	Mobile phase	MS	Analytes *	LOD and LOQ	Linear Range	Reference
Postmortem whole blood	MeOH, SPE	PC(Phosphorychloline)–HILIC	1% AA + ACN in MeOH	LC-MS/MS	6 and voglibose	LOD: 0.32 ng/mL LOQ: 1.08 ng/mL	2–1200 ng/mL	[104]
Urine and plasma	2% AA, C_18_ and ZIC-HILIC SPE	Atlantics dC_18_	10 mM AF + FA (95:5, v/v) + 5 mM HFB in 2% ACN	LC-MS/MS	6	LOD: LOQ:	10–500 ng/mL	[135]
Blood and urine	C_18_ and Oasis MCX SPE	Allsphere ODS-2 (LC-UV) Nova-Pak C_18_ (LC-LFD) Zorax 300SB-C_3_ (LC-MS/MS) HILIC (LC-MS/MS) Atlantics dC_18_ (LC-MS/MS)	4.8 mM 1-HSA + 41.8 mM SDP + 10% MeOH, pH (Allsphere ODS-2) 5 mM PIC B7 (HSA) + 3% MeCN in H_2_O, pH 4.5 (Nova-Pak C_18_) 10 mM TMA, 10 mM AF in 1% ACN, pH 4 (Zorax 300SB-C_3_) 0.1% FA in MeOH (HILIC) 10 mM AF + FA, (95:5, v/v) + 5 mM HFB + 2% ACN	LC-UV LC-LFD LC-MS/MS LC-MS/MS LC-MS/MS	6	LOD: 10 ng/mL (LC-UV) LOQ: 5 and 20 ng/mL for serum and urine (LC-LFD) LOD: 15.6 nM (LC-MS/MS) LOD: 0.1 ng/mL LOQ: 1 ng/mL (LC-MS/MS) LOD: 0.13 ngmL^−1^ LOQ: 2.5 ngmL^−1^ for urine and plasma	10–50,000 ng/mL (LC-UV) 20–300 for urine and 5–20 ng/mL for serum (LC-LFD) 93.75–9375 nM (LC-MS/MS) 1–100 ng/mL 0–500 ngmL^−1^ for urine and 0–20 ngmL^−1^ for plasma	[13,106,135,196]
Cooked and raw puffer fish (liver) and human urine	1% AA in MeOH	TosoHaas TSK-GEL Amide-80	5 mM AF + 26.5 mM FA in ACN: H_2_O, 70:30	LC-MS/MS	6	20 µg/100g tissue	1–10,000 ng/mL	[136]
Urine and blood	0.5 M AA, C_18_ SPE	Zorax 300SB-C_3_	1% ACN + 10 mM TMA + 10 mM AF, pH 4	LC-MS	6	LOD: 15.6nM	93.75–9375 nM	[106]
Std mixture	Not used	TSKgel Amide-80	16 mM AF, pH 5.5 + ACN (3:7, v/v)	LC-MS/MS	6, 7, 14, 22	NR	64 pg–2 ng 64 pg–2 ng 128 pg–1 ng 180 pg–1.4 ng	[128]
Serum	0.5 M AA in MeOH, Oasis MCX SPE	Cosmosil HILIC 4.6 × 150 mm	0.1% FA in water + MeOH	LC-MS/MS (M. Horie *et al*., 2002)	6, 7, 16	LOD: 0.1 ng/mL LOQ: 1 ng/mL	1–100 ng/mL	[13,100]
Urine and serum	Urine Extraction: C_18_ Sep-Pak SPE (0.2 M HCl in 20% MeOH) followed by Strata X-C 33 µm Cation Mixed-Mode Polymer SPE (0.1 M HCl+MeCN+MeOH+Water) Serum Extraction: Oasis MCX SPE (0.2 M HCl in 20% MeOH +MeCN+MeOH+Water)	Nova-Pak C_18_ 4 µm, 8 × 100 mm	PIC B7 (Heptane sulfonic acid), 5 mM + 3% MeCN, pH 4.5 (adjusted with conc. NH_3_)	LC-FLD	6	LOD: 20 ng/mL (Urine) 5 ng/mL (Serum) LOQ: 20 ng/mL (Urine) 5 ng/mL (Serum)	20–300 ng/mL (Urine) 5–20 ng/mL (Serum)	[100]

Note: (Analyte*) Refer to Table 3 for the name of the TTX analytes.

**Table 10:** Recovery of TTX from different matrices.

Matrix	Extraction Method	% Recovery	Reference
Trumpet shell	ASE (Accelerated solvent extraction) and SE (Solvent Extraction) (0.03 M AA) (UPLC–MS/MS)	80–92	[48]
Gastropod tissue	1% AA in MeOH, C18-SPE, ultrafiltration (<3000 MW), (HPLC-FLD)	90	[13]
Xanthid crab, *Demania cultripes*	1%AA in MeOH, C18-SPE, ultrafiltration (<3000 MW), (LC-MS)	86.3 ± 2.9	[139]
Puffer fish ovary	0.05M AA, ODS-SPE, ultrafiltration (0.22 µ), (LC-MS)	94.2–9108.3	[127]
Puffer fish tissues, Muscle, Skin and Liver	2% AA, methacrylate-styrene divinyl benzene cartridge (LC-MS) C18 column (50 mm × 2.1 mm i.d.) using 10 mmol/L IPCC-MS7-methanol (65:35) as the mobile phase at a flow rate of 0.2 mL/min	Muscle 79–83 Skin 85–88 Liver 85–90 (LOD 0.01 µg/g tissue)	[244]
Puffer fish eggs and newt	0.1% AA, Cosmosil 75C18-OPN resin-SPE, CHCl_3_ wash, (LC-MS)	>90	[175]
Newt (Whole body)	0.1% AA in 70% MeOH, charcoal adsorption, (HPLC-FLD)	50	[35]
Blood serum	0.5 M AA and Oasis MCX-SPE, ultrafiltration (<3000 MW) (LC-MS/MS)	>95	[13]
Whole Blood	1% AA in MeOH, PCX-SPE, (LC-MS/MS)	TTX 61.4Voglibose 62.8	[104]
Human urine and plasma	C-18 and HILIC SPE (LC-MS/MS)	75–81	[135]
Human urine and blood	0.5 M AA, C18 SPE, ultrafiltration (<3000 MW), (LC-MS)	Urine 90.9 ± 1.4 Blood 90.6 ± 0.2	[106]
Human urine and blood	2% AA, methacrylate-styrene divinyl benzene cartridge (LC-MS) C18 column (50 mm × 2.1 mm i.d.) using 10 mmol/L IPCC-MS7-methanol (65:35) as the mobile phase at a flow rate of 0.2 mL/min	Human serum 93–96 (0.1 ng/mL) Human urine 93-101 (0.1 ng/mL)	[244]
Combined muscle, liver and ovary from tiger puffers and muscle and ovary from balloon fishes	1% AA in MeOH, defatted with chloroform (HPLC-FLD)	91.0 ± 5.2	[43]
Puffer fish muscle, liver and phosphate buffered saline	1% AA in MeOH, defatted with chloroform (HPLC-FLD)	86.4 ± 18.9	[136]

Several extraction studies have been conducted to improve the recoveries of TTX in a number of sample types. Fong *et al*., 2011 [135] stated that acetonitrile and methanol give similar recoveries and in view of the global shortage of acetonitrile and consequently its increased expense the preferred choice must be methanol. The same group found that non-acidified solutions tend not to retain TTX so they used 2% acetic acid to enhance retention. In most of the studies conducted, scientists have used between 0.1 to 1% acetic acid for the extraction of TTX from different types of matrices (Table 4, Table 5, Table 6, Table 7, Table 8 and Table 9) and defatting of samples was usually accomplished with either chloroform, hexane or dichloromethane followed by charcoal adsorption for better recovery. 

Many methods for the extraction of the TTXs include a solid phase extraction (SPE) step using C-18 cartridges and/or Bio-gel filtration for sample clean up prior to analysis. Recently, accelerated solvent extraction (ASE) was applied for the extraction of TTX from puffer fish and trumpet shell by Nzoughet *et al*., 2013 [48]. ASE gave better recoveries for the extraction of TTX from trumpet shell [48]. But the sample needs to be lyophilized (freeze-dried) which make it a time consuming process. Fong *et al*., 2011 [135] state that HILIC SPE should be carried out under gravity flow as the application of a vacuum compromises the recovery of TTX.

#### 11.3.2. Development in Chromatography

Reverse phase chromatography was used for many years for the analysis of TTX and its analogues (most commonly C_18_). But all the analogues of TTX could not be separated using reverse phase chromatography. Others tried normal phase chromatography for the separation of the TTXs (most commonly TSK-GEL Amide-80 and HILIC). TTX is a polar compound and thus it retains quickly on reverse phase columns, but retains slowly on normal phase columns, also giving better separation of its analogues.

HILIC (Hydrophilic interaction liquid chromatography) column has hydrophilic stationary phase operated using reversed-phase type eluents; while ZIC-HILIC (Zwitter ionic hydrophilic interaction liquid chromatography) column has densely bonded, zwitter ionic functional groups. Separation is achieved by hydrophilic partitioning combined with weak ionic interactions for maximum selectivity, high load ability and easy optimization of methods. ZIC-HILIC columns are very efficient to improve peak resolution for polar and hydrophilic compounds. 

Nakagawa *et al*., 2006 gave a brief description of the development of LC-MS for the detection of the TTXs [175]. They detected 11-deoxyTTX and 5,6,11-trideoxyTTX using a HILIC chromatography [175].

Chen *et al*., 2011 developed and validated a method for separation of TTX analogues from puffer fish [127]. They used an Atlantis HILIC Silica column (100 × 2.1 mm, 3 µm) with which they achieved a total run time of 10 min. Yotsu-Yamashita *et al*., 2011 could separate TTX from 4-epiTTX using a HILIC column. However 4,9-anhydro-5,6,11-trideoxyTTX and 4,4a-anhydro-5,6,11-trideoxyTTX were not clearly separated by HILIC chromatography [128]. In addition 5,6,11-trideoxyTTX and 8-epi-5,6,11-trideoxyTTX were not resolved using a HILIC chromatography, their determination was hindered from not only from sharing the same retention time but also by producing an identical MS/MS fragmentation pattern [59]. Of course the elution profile of the analogues of TTX on polar chromatography is opposite to that found in reverse phase chromatography [29]. A very good separation of TTX and its analogues was achieved using a ZIC-HILIC (5 µm, 150 × 2.1 mm) column from the extracts of *Takifugu oblongus* [138].

The most frequently applied mobile phases for TTX analysis were 10 mM ammonium formate + 10 mM formic acid in H_2_O and acetonitrile; 5 mM ammonium heptafluorobutyrate + 50 mM ammonium acetate or ammonium formate, pH 5 in 3% acetonitrile for puffer fish, gastropod, newt, bacteria and human urine and blood; 2 mM sodium 1-heptane sulfonate in 1% Methanol + K_3_PO_4_, 0.05 M, pH 7 and 3% Acetonitrile + 0.97% heptafluorobutyric acid + 0.29% AA, pH 5 for gastropod and newt. And 30 mM heptafluorobutyric acid + 1 mM ammonium acetate was often used as the mobile phase for the analysis of TTX in crab and frog samples. 

The LC-MS methods that have been used for TTX analysis have been reviewed comprehensively by Leung *et al*., 2011 (they determined TTX levels using LC-MS in the urine and plasma of Asian patients) [196]. They brought total chromatographic run time to 5.5 min using an Atlantics dC_18_ (2.1 mm × 150 mm, 5 µm) column with flow rate of 200 µL min^−1^. This method was validated and applied on human urine and blood matrices for the detection of TTX [135]. They also studied the effect of an ion pair reagent (heptafluorobutyric acid) and optimized the concentration of the ion pair reagent at 5 mM. They found significant ion suppression with the MCX-HILIC, MAX-HILIC and HLB-HILIC columns but not with the C_18_-HILIC and Sep-Pak-HILIC. Finally they used C_18_-HILIC as it gave the most intense peaks. 

The best reported LODs for TTX to-date are 0.074 ng/mL (0.23 nM) [48] in puffer fish and trumpet shell samples; 0.7 pmol (0.00022 ng/mL) at S/N 2 [149] in puffer fish samples; 0.4 pmol (0.00013 ng/mL) in newt samples [149]; 0.005 µg/mL (15.67 nM) in crab samples [139] and 0.1 ng/mL (0.31 nM) in human urine and plasma samples [13].

#### 11.3.3. Development in Mass Spectrometry

The fragmentation patterns of TTX had been studied extensively by many research groups. However the fragmentation patterns of the analogues of TTX have not been not widely investigated. TTX gives characteristic fragments: *m*/*z* 302 (representing the loss of 1 water molecule), 284 (representing the loss of 3 water molecules), and at *m*/*z* 256, 178 and 162 [27,29,34,45,128,149]. 6-epiTTX gives characteristic fragments at *m*/*z* 302, 284, 256, 178 and 162 [149]. 4-epiTTX, 11-norTTX-6-(S)-ol, 4,9-anhydroTTX, 5-deoxyTTX and 5,6,11-trideoxyTTX have a common fragment at *m*/*z* 162 [128,149]. 11-norTTX-6-(S)-ol and 11-norTTX-6,6-diol both give a fragment at *m*/*z* 178 [149]. 5-deoxyTTX and 11-deoxyTTX give a characteristic fragment at *m*/*z* 176 [149]. 5-deoxyTTX and 5,6,11-trideoxyTTX give characteristic fragment at *m*/*z* 146 [149]. 11-oxoTTX gives a characteristic fragment at *m*/*z* 336, 318 (loss of 1 water molecule), 300 (loss of 2 water molecules), 282 (loss of 3 water molecules), and at *m*/*z* 178 and 162 [34].

Rodriguez *et al*., 2008 [45] suggests that the formation of the TTX MS ion at *m*/*z* 256 could be due to elimination of CO at C-10 causing cleavage of the bond between C-9 and C-10, C-10 and C-5-O and C-10 and C-7-O. The group also suggest that the formation of ion at *m*/*z* 254 in 5,6,11-trideoxy-TTX is due to the loss of one water molecule from the original structure. Fragment ions at *m*/*z* 162 and 178 are assigned as 2-aminohydroxyquinazoline and 2-aminodihydroxyquinazoline respectively which may be formed by the breaking of bonds between C-8a and C-9 and between C-6 and C-11 [149].

Some researchers have discovered unknown compounds that possess similar fragmentation patterns to TTX which may suggest the presence of unknown analogues of TTX in species known to contain TTX. Pires Jr. *et al*., 2005 [72] have found unknown compounds in *Brachycephalus* sp. with fragment ions at *m*/*z* 330 and *m*/*z* 348 which are also present in the MS/MS spectra of TTX; they have also identified ions at *m*/*z* 162 (2-aminohydroxyquinazoline) and *m*/*z* 178 (2-aminodihydroxyquinazoline) in those unknown compounds. There is wide scope for further investigations of fragmentation patterns of TTX analogues. Some of the analogues of TTX share the same mass and fragmentation pattern (two most intense ions) suggesting the need for full chromatographic resolution in studies relating to TTX.

#### 11.3.4. Quantitative TTX Analysis

In most of the TTX studies, TTX analogues were quantified against a standard calibration curve of TTX, because certified standards for the analogues of TTX are commercially unavailable. In some studies, concentrations of TTX analogues have been calculated against standard calibration curves of individual analogues: for 4-epi TTX, 4,9-anhydro TTX and 5,6,11-deoxyTTX with good correlation regression coefficents (r^2^ = 0.99) obtained [128]. Kudo *et al*., 2012 calculated the concentration of 5,6,11-trideoxyTTX in samples against a standard curve of purified 5,6,11-trideoxyTTX [59]. 

**Figure 6:**
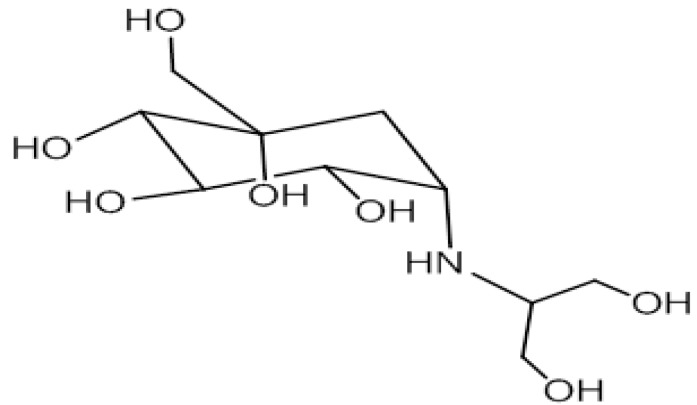
Structure of voglibose (Internal standard for TTX).

In many studies TTX itself was used as the internal standard for the quantitation of its analogues [127]. Cho *et al*., 2012 [104] has used voglibose (*m*/*z* 268/92) as an internal standard for TTX (Figure 6). They used voglibose as an internal standard because isotope labelled TTX was not commercially available. Voglibose has a similar structure that of TTX and also had similar chromatographic and MS properties, so it is a good IS for TTX analysis. Man *et al*., 2010 [137] used salicylic acid as the internal standard. 

#### 11.3.5. Matrix Effect in Puffer Fish, Trumpet Shell and Human Urine/Blood Sample

Ion suppression or matrix effects in LC-MS and LC-MS/MS is the reduction in detector response, or signal: noise produced by analytes of interest due to the competition for ionisation, between the analyte of interest and other compounds present in the sample matrix (e.g., polymers from plastic tubes and filters used for extraction, mobile phase additives *etc*.) which have not been removed from the sample matrix during extraction. Ion suppression effect reduces sensitivity of analysis if ion suppressing compounds co-elute with the analytes of interest. Therefore, it is very important to remove all the impurities from sample matrix [245]. 

Matrix effects in puffer fish, monkfish and human urine samples were compared between SPR and LC-MS/MS methods, however much of the detail was omitted from the publication [136] Tsai *et al*., 2006 [106] found that the levels of TTX were higher in urine than in blood samples 10 h after TTX ingestion. So urine is a better choice of sample for confirming TTX poisoning. Urine is a complex matrix containing many polar compounds therefore when using HILIC chromatography ion suppression caused by matrix components may compromise quantitation [196]. The ion suppression effect (post column infusion study), precision and accuracy (*n* = 10) was conducted for urine and plasma samples by Fong *et al*., 2011 [135]. They used double SPE (C-18 followed by ZIC-HILIC SPE) for better recovery of TTX. Cho *et al*., 2012 [104] obtained 98.3%–111.2% average matrix effect in blood when using the internal standard, voglibose (Figure 6) for their study.

## 12. Measures to Ensure Human Safety (Legislation)

Table 11 summarizes toxicity limits of TTX and some of its analogues. Acceptability limit in puffer fish as food in Japan is 10 MU TTX eq/g or 2.2 µg TTX eq/g of puffer flesh [131]. Regulatory limit for TTX in food is 2000 µg/kg TTX eq [246] while in the US a zero level of TTX in food exists [247]. But as it is newly emerging toxin in Europe, regulatory limits for TTX in food are still not established. There is a need of setting proper regulatory limits for TTX to ensure food safety.

**Table 11:** Toxicity limits for TTX.

Description	Value	Reference
Human median lethal dose	8.7 µg/kg	[127]
MLD for mammals (IP or IV)	2.7–10 µg/kg for rats 4.5 µg/kg for guinea pigs 8–10 µg/kg for mice, rabbits, dogs and cats	[97]
Lethal potency	5,000–6,000 MU/mg	[46]
MLD for human	10,000 MU (≈2 mg)	[46]
Regulatory limit in food in US	Zero	[247]
LD_50_ of TTX in mice	9 µg/kg	[19]
LD_99_ of 5,6,11-trideoxy TTX	750 µg/kg	[124]
IC_50_ for nine human functional voltage-gated Na^+^ channels	≥1 μM	[97]
MLD of TTX to humans	2 mg/50 kg BW	[131]
Minimum acute dose of TTX to humans	0.2 mg/50 kg BW	[131]
Acceptability limit in puffer fish as food in Japan	10 MU TTX eq/g or 2.2 µg TTX eq/g of puffer flesh	[131]
LD_50_ of TTX in mice, dogs and rabbits	8–14 µg/kg by injection	[76]
Lethal dose	2 mg	[25]
LD_50_ of TTX	10 µg/kg (IP in mice)	[129]
LD_50_ of 11-deoxy TTX	70 µg/kg (IP in mice)	[31]
IC_50_ for 6,11-dideoxy TTX	420 µg/kg (IP in mice)	[125]
Regulatory limit in food in Japan	2000 µg/kg TTX eq	[246]
Lethal doses in KM mice	LD_1_: 9.4 µg/kg LD_50_: 11.3 µg/kg LD_99_: 13.5 µg/kg	[130]

## 13. Conclusions

In areas where TTX occurs with regularity, it is important that rapid analytical methods are deployed for the analysis of clinical samples, most especially blood and urine in suspected poisoning victims. LC-MS/MS methodologies are particularly appropriate to detect TTX and its analogues in clinical samples with the speed required in such cases. Though there is still no commercially available antidote to TTX, it may dictate the course of medical treatment, especially for those with compromised renal function.

There is hope that in the future an antidote may be developed to counteract the effects of the toxin *in vivo*. In the meantime more research is required on the prevalence and the toxicity of TTX metabolic bioconversion products in vector species in order to develop a comprehensive human risk analysis. 

A report in the wildlife section of “The Times” newspaper dated 11 May 2013 [248] revealed that a red scorpion fish had been caught in the Celtic Sea off the coast of Ireland and England. This is clear evidence that exotic and toxic marine species can travel and may be commonly found in cooler European waters in the future. If migration trends like this are to continue and there are predictions that they are likely (in view of global warming), it may be prudent to carry out surveillance of susceptible marine species, algae and seawater in European territory for TTX and other toxins associated with warmer regions.

## Acronyms

4-AP: 4-aminopyridine
AA: Acetic acid
ACN: Acetonitrile
AF: Ammonium formate
AHB: Ammonium heptafluoro butyric acid
Am-acetate: Ammonium acetate
Am-OH: Ammonium hydroxide
ASE: Accelerated solvent extraction
BW: Body weight
CharAd ^†^: Charcoal adsorption
CID: Collision induced dissociation
DCM: Dichloromethane
Defat *: Defatting
ELISA: Enzyme linked immunosorbent assay
Eq: Equivalent
EtOH: Ethanol
FA: Formic acid
GC-MS: Gas chromatography- Mass spectrometry
GTX: Gonyautoxin
HCD: High collision induced dissociation
HFB: Heptafluoro butyric acid
HLB-HILIC: Hydrophilic-lipophilic balance- HILIC
HILIC: Hydrophilic interaction liquid chromatography
HPLC: High performance liquid chromatography
HAS: Heptane sulfonic acid
IC_50_: Half maximal inhibitory concentration is a measure of the effectiveness of a compound in inhibiting biological or biochemical function
IP: Intraperitoneal
IR: Infrared resonance
IV: Intravenous
LC-FLD: Liquid chromatography-fluorescent detection
LC-MS: Liquid chromatography-Mass spectrometry
LC-PDA: Liquid chromatography-Photo diode array detection
LC-UV: Liquid chromatography-Ultra violet detection
LD_50_: Lethal dose_50_ of a toxin is the dose required to kill 50% of the members of a tested population after a specified test duration.
LD_99_: Lethal dose_99_ of a toxin is the dose required to kill 99% of the members of a tested population after a specified test duration.
LOD: Limit of detection
LOQ: Limit of quantitation
MAX-HILIC: Mixed-mode anion exchange- HILIC
MCX-HILIC: Mixed-mode cation exchange- HILIC
MeCN: Methyl cyanide
MeOH: Methanol
MLD: Median lethal dose
MRM: Multiple reaction monitoring
MU: Mouse unit; 1 MU is defined as the amount of toxin required to kill a 20 g ICR (Institute of Cancer Research) strain mouse in 30 min after intraperitoneal injection [81]
MW: Molecular weight
*m*/*z*: mass/charge ratio
NMR: Nuclear magnetic resonance
PSP: Paralytic shell fish poisoning
Q-TOF MS: Quadrupole-time-of-flight mass spectrometry
SE: solvent extraction
SIR: Selected ion recording
S/N: Signal/Noise ratio
SPE: Solid phase extraction
STX: Saxitoxin
TBA-PO_4_: Tetrabutyl ammonium phosphate
TLC: Thin layer chromatography
TMA: Trimethyl amine
Tris-Ac: Tris- acetate buffer
TTX: Tetrodotoxin
UPLC: Ultra performance liquid chromatography
ZIC-HILIC: Zwitter ionic hydrophilic interaction liquid chromatography
